# Strategies for inducing diabetes in laboratory animals: Advances from chemical, surgical, immunologic, dietary, and genetic approaches

**DOI:** 10.1002/ame2.70259

**Published:** 2026-07-30

**Authors:** Milad Faraji, Maedeh Faraji, Nasrin Kazemipour

**Affiliations:** ^1^ Department of Basic Sciences, School of Veterinary Medicine Shiraz University Shiraz Iran; ^2^ Department of Biology, College of Sciences Shiraz University Shiraz Iran

**Keywords:** CRISPR, diabetes mellitus, experimental modeling, in vivo, laboratory animal

## Abstract

Diabetes mellitus is a metabolic disorder characterized by hyperglycemia resulting from defects in insulin secretion, insulin action, or both. It involves complex metabolic, hormonal, and immunological mechanisms that cannot be fully reproduced in vitro, making in vivo animal models essential for mechanistic and translational research. This review provides a comprehensive overview of strategies used to induce diabetes in laboratory animals. The principal approaches are categorized as follows: (i) chemical induction (e.g., streptozotocin, alloxan, dithizone), (ii) dietary and hormonal interventions (e.g., high‐fat diets, glucocorticoids), (iii) immune‐ and virus‐mediated models (e.g., Coxsackievirus‐triggered diabetes), (iv) surgical models (e.g., partial pancreatectomy), and (v) genetically engineered or spontaneously diabetic strains (e.g., NOD mice, GK rats, db/db mice). For each category, we describe the underlying mechanisms, typical protocols (including doses, duration, and onset of hyperglycemia), and their relevance for modeling type 1 or type 2 diabetes. Although chemical models are widely applied because of their technical simplicity, reproducibility, and relatively low cost, they may not fully replicate autoimmune or progressive metabolic features of human disease. Comparative tables summarize model characteristics, including mechanisms, strengths, limitations, disease onset, and research applications, to facilitate informed model selection. No single animal model fully reproduces the species‐specific immune complexity, progressive metabolic deterioration, and long‐term complications observed in human diabetes. Nevertheless, these models provide controlled and mechanistically tractable systems for investigating β‐cell dysfunction, insulin resistance, inflammatory pathways, and therapeutic interventions. Careful alignment between model characteristics and research objectives—and, when appropriate, the complementary use of multiple models—is therefore essential to enhance translational relevance.

## Graphical abstract

This review summarizes the principal experimental approaches used to induce diabetes in animal models. Strategies include chemical agents (streptozotocin, alloxan, dithizone, gold thioglucose), dietary interventions (high‐fat and high‐sugar diets), surgical methods (total or partial pancreatectomy), genetic models (db/db, ob/ob, Goto‐Kakizaki [GK], Otsuka Long‐Evans Tokushima Fatty [OLETF], KK/Ay, BioBreeding), immunological approaches (nonobese diabetic [NOD], antigen based, checkpoint inhibitors), viral induction (encephalomyocarditis virus [EMCV], Coxsackievirus B, rotaviruses), and hormonal manipulations (glucocorticoids, growth hormone [GH], glucagon, sex steroids). Each method reflects distinct mechanisms and phenotypes, enabling researchers to mimic type 1 or type 2 diabetes. Selection of the appropriate model depends on the study objectives and the targeted pathophysiological features of diabetes.

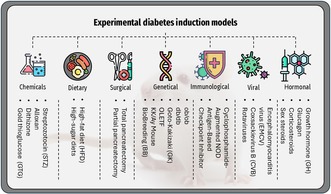

## 1 INTRODUCTION

Diabetes mellitus is a multifactorial metabolic disorder characterized by hyperglycemia due to defects in insulin secretion, action, or both. It is associated with long‐term damage to multiple organ systems and represents a major and rapidly growing global health challenge.^1^ According to the International Diabetes Federation, the number of individuals with diabetes is projected to increase by 46% between 2021 and 2045, reaching approximately 783 million worldwide.^2^

The International Diabetes Federation classifies diabetes mellitus into three principal categories: type 1, type 2, and gestational diabetes. These forms differ in etiology, pathophysiology, and clinical presentation.

Type 1 diabetes (T1D) is an autoimmune disorder characterized by T‐cell–mediated destruction of pancreatic β cells, leading to absolute insulin deficiency and chronic hyperglycemia 3. It typically manifests in childhood or adolescence, although adult onset may occur.^3^

Type 2 diabetes (T2D) is defined by a combination of peripheral insulin resistance and relative insulin deficiency. It is strongly associated with obesity, physical inactivity, aging, and genetic susceptibility and accounts for the majority of global diabetes cases. Patients with T2D are at increased risk for both microvascular and macrovascular complications.^4^

Gestational diabetes mellitus (GDM) refers to glucose intolerance first recognized during pregnancy and is primarily driven by pregnancy‐associated insulin resistance in genetically or metabolically predisposed women.^5^ Although often transient, GDM increases the long‐term risk of developing T2D in both mother and offspring.^5^

Together, these distinct but overlapping forms of diabetes provide important frameworks for the development and selection of experimental animal models.

Animal models play a pivotal role in elucidating the pathogenesis of diabetes and in supporting the preclinical evaluation of therapeutic strategies. Compared to in vitro systems, in vivo models enable dynamic investigation of hormonal regulation, metabolic interactions, and disease progression within a whole‐organism context.^6^ Rodents—particularly rats and mice—remain the most widely used species because of their short reproductive cycles, genetic tractability, manageable size, and cost‐effectiveness.^7^, ^8^, ^9^ However, important limitations must be considered, including species‐specific genetic and physiological differences that may affect translational relevance, as well as the influence of controlled laboratory environments on metabolic phenotypes and disease progression. Additionally, differences in immune system architecture and β‐cell biology between rodents and humans may further limit direct extrapolation of findings to clinical settings.^10^, ^11^, ^12^, ^13^

This review outlines the major mechanistic categories used to induce diabetes in laboratory animals. These include chemical, dietary and metabolic, surgical, genetic and spontaneous, immune‐mediated, viral, and hormonal models, each representing distinct and well‐characterized pathogenic mechanisms relevant to either T1D or T2D. The following sections systematically analyze these approaches, their mechanisms, induction protocols, utilities, and limitations.

Accurate interpretation of experimentally induced hyperglycemia requires a species‐specific physiological context, an aspect often overlooked in previous reviews. Baseline fasting blood glucose (FBG) varies considerably across laboratory animals, yet veterinary physiology references typically report only normal fasting ranges rather than categorical glycemic states used in human medicine. These reference intervals are generally derived from measurements obtained in clinically healthy, age‐ and strain‐appropriate animals following standardized fasting periods and are widely used as physiological benchmarks for evaluating glucose homeostasis. To enhance experimental reproducibility and cross‐study comparability, we compiled established reference intervals for fasting glucose across commonly used species—including mice, rats, rabbits, hamsters, dogs, cats, domestic pigs, minipigs, and zebrafish (Table 1).

**TABLE 1:** Normal fasting blood glucose reference intervals in commonly used laboratory animal species.

Common name	Species	Strain/type	Normal fasting glucose (mg/dL)	References
Mouse	*Mus musculus*	*–*	~72–105	[14]
Rat	*Rattus norvegicus*	Wistar	~70–117	[15]
		Sprague–Dawley	~80–145	[16]
Rabbit	*Oryctolagus cuniculus*	New Zealand White	~75–155	[17]
Hamster	*Mesocricetus auratus*	Syrian	~32.6–118.0	[18, 19]
Dog	*Canis lupus familiaris*	Domestic dog	~76–119	[17]
Cat	*Felis catus*	Domestic cat	~60–120	[17]
Pig	*Sus scrofa domesticus*	Domestic pig	~85–150	[17]
		Minipig (Göttingen)	~40–80	[20]
Zebrafish	*Danio rerio*	–	~50–80	[21, 22, 23, 24]

Although several review articles have previously summarized experimental models of diabetes,^25^ including the comprehensive overview by Singh et al.,^26^ many earlier works remain limited in scope—focusing primarily on rodent models, describing induction strategies in isolation rather than in an integrated mechanistic framework, or lacking species‐specific physiological baselines critical for interpreting hyperglycemia across animal models. Moreover, previous reviews seldom harmonize fasting glucose reference ranges across species, despite the central role of baseline glycemia in assessing the success, severity, and reproducibility of diabetes induction protocols. Species‐specific baseline values are also essential for selecting appropriate experimental controls and for avoiding misinterpretation of hyperglycemia when comparing findings across different animal models.

The present review aims to address these gaps by (i) integrating chemical, dietary, metabolic, surgical, genetic, immune‐mediated, viral, and hormonal models into a unified comparative framework; (ii) expanding the taxonomic breadth to include rabbits, dogs, cats, domestic pigs, minipigs, and zebrafish; (iii) incorporating advances from emerging translationally relevant systems, including genetically engineered and CRISPR‐based models; and (iv) providing standardized species‐specific fasting glucose intervals to improve methodological consistency, reporting quality, and translational relevance. Together, these additions offer a more comprehensive, mechanistically coherent, and physiologically contextualized synthesis than previous reviews, thereby providing investigators with a robust decision‐making framework for model selection in diabetes research.

## 2 EXPERIMENTAL STRATEGIES FOR INDUCING DIABETES

### 2.1 Chemical induction methods

Various chemical agents have long been used to induce diabetes in experimental models by selectively damaging pancreatic β cells. Among these, streptozotocin (STZ) and alloxan are the most widely used compounds, enabling researchers to investigate the physiological changes associated with the onset and progression of diabetes. Due to their ease of use, reproducibility, and rapid induction of hyperglycemia, chemical methods remain the most widely adopted approach in experimental diabetes research.^27^, ^28^, ^29^, ^30^

#### 2.1.1 Streptozotocin

Streptozotocin (also called streptozocin) or 2‐deoxy‐2(([methyl(nitroso)amino]carbonyl)amino)‐(α and β)‐d‐glucopyranose^31^ was first extracted from *Streptomyces achromogenes* in 1960, although its diabetogenic properties were not identified until 1963.^32^ STZ is a widely adopted chemical agent for diabetes induction due to its superior reproducibility and stability compared to alloxan.^26^ A single high dose of STZ (e.g., 65–150 mg/kg in rats or ≥ 150 mg/kg in mice) causes near‐complete β‐cell ablation within days, generating an insulin‐dependent T1D‐like phenotype.^33^, ^34^ STZ has been employed across various species, including rodents (mice and rats), dogs, pigs, and others. However, its effectiveness and toxicity vary depending on factors such as age (with younger animals being more sensitive), sex (males typically more sensitive than females), genetic strain (e.g., Wistar and Sprague–Dawley being highly sensitive, WKY less so), and nutritional status. Intravenous (IV) administration generally leads to more consistent hyperglycemia than intraperitoneal (IP) injection.^35^ For optimal stability, STZ must be freshly prepared or allowed to dissolve for 1–2 h before use, and appropriate protective measures are required due to its mutagenic and oncogenic potential.^26^, ^34^, ^35^

To prevent early STZ‐induced hypoglycemia, some protocols incorporate glucose supplementation or dose reduction. Multi‐low‐dose STZ (MLD‐STZ, 20–40 mg/kg/day for 5 consecutive days) induces more gradual β‐cell loss and elicits immune‐mediated responses that closely resemble autoimmune T1D.^36^, ^37^, ^38^ Cyclophosphamide, an alkylating chemotherapeutic agent, has also been combined with MLD‐STZ to accelerate autoimmune diabetes onset. A single dose of cyclophosphamide (e.g., 200 mg/kg), with or without STZ, can break immune tolerance by simultaneously damaging β cells and enhancing the activity of autoreactive T cells.^26^ These immune‐potentiating protocols are widely used to study autoimmune mechanisms (see “Immune Models”).

A notable advancement is the use of STZ to model T2D. When coadministered with nicotinamide (NA), a β‐cell–protective agent, or given at lower doses following a high‐fat diet (HFD), STZ produces partial β‐cell impairment alongside insulin resistance—closely simulating late‐stage T2D. Nicotinamide, a precursor of NAD^+^, inhibits poly(ADP‐ribose) polymerase activity and thus mitigates STZ‐induced cytotoxicity.^39^ Pretreating rodents with NA (e.g., 90–230 mg/kg IP) 15–30 min before a moderate STZ dose (40–65 mg/kg) allows partial β‐cell survival. These NA–STZ models typically exhibit mild‐to‐moderate hyperglycemia with residual insulin secretion.^40^ For instance, rats receiving STZ (65 mg/kg) plus NA (90 mg/kg) gradually develop hyperglycemia, insulin resistance, and T2D‐like phenotypes over several weeks.^41^, ^42^ In contrast, administration of STZ alone at similar doses results in near‐total β‐cell destruction and T1D‐like features.^40^ The NA–STZ model is now extensively used for T2D research due to its rapid induction, cost‐effectiveness, and independence from obesity. In this model, NA partially protects β cells by elevating intracellular NAD^+^ levels and enhancing nitric oxide production, counteracting the DNA‐damaging effects of STZ. The remaining β cells can initially maintain glucose regulation but eventually fail under insulin‐resistant conditions.^39^ The severity of the diabetic phenotype in this model depends on the dose and timing of NA relative to STZ administration, with most protocols delivering NA 15–30 min prior to STZ.

This model offers several advantages, including reproducible moderate hyperglycemia (typically 200–300 mg/dL), preserved insulin secretion resembling human T2D, and the absence of obesity‐related confounding factors. However, it also has limitations: it does not replicate obesity‐driven insulin resistance, and β‐cell loss is chemically induced rather than emerging through natural disease progression.

STZ is also widely and reliably used in large‐animal models. In minipigs, its diabetogenic effects closely parallel human T1D. Long‐term studies in Wuzhishan miniature pigs demonstrate sustained hyperglycemia, β‐cell destruction, and chronic complications such as cataracts, retinopathy, and nephropathy, with occasional partial β‐cell regeneration.^43^ Göttingen minipigs develop stable insulin‐dependent diabetes following STZ (IV), showing marked β‐cell loss and impaired glucose tolerance, making them suitable for long‐term complication and therapy studies.^44^, ^45^

STZ‐based T2D models have also been established in porcine species. Lee et al.^46^ showed that NA‐modulated STZ induction in micropigs results in partial β‐cell destruction, moderate hyperglycemia, and insulin deficiency, functionally paralleling rodent NA–STZ protocols and offering a translationally relevant large‐animal platform. Collectively, these findings underscore the value of minipigs and micropigs in bridging rodent research and human diabetes studies.

In nonhuman primates, STZ (10–40 mg/kg IV) induces significant β‐cell impairment, raising fasting glucose from <70 to >400 mg/dL.^47^ A single high dose (~100 mg/kg IV) produces stable insulin‐deficient diabetes in cynomolgus and rhesus macaques, with complete C‐peptide loss and ~10% transient nephrotoxicity.^48^ Lower fractionated dosing is less reliable and increases hepatic and renal risk. High‐dose pharmaceutical‐grade STZ therefore remains the standard for generating robust T1D‐like primate models.

STZ is also used to induce T1D in Syrian golden hamsters. Repeated IP injections (40–50 mg/kg for 3 days) cause profound β‐cell loss, hyperglycemia (>300 mg/dL), ~50% insulin reduction, and persistent diabetic dyslipidemia.^49^ Microvascular abnormalities appear within 2 months, making this a useful small‐animal T1D model.

Adult zebrafish likewise develop STZ‐induced diabetes. A single IP injection of 350 mg/kg STZ results in sustained hyperglycemia (~300 mg/dL vs. ~60 mg/dL baseline). Due to rapid β‐cell regeneration in zebrafish, weekly booster doses are required to maintain hyperglycemia for up to 90 days. These models exhibit T1D‐like features, including reduced circulating insulin, diminished islet insulin staining, elevated glycated proteins, and early nephroretinal pathology.^50^

#### 2.1.2 Alloxan

Alloxan, first identified as a diabetogenic compound in 1942,^9^, ^51^ is an organic urea derivative (5,5‐dihydroxyl pyrimidine‐2,4,6‐trione) that functions as a cytotoxic glucose analog with reported carcinogenic potential.^52^ Like STZ, it is transported into pancreatic β cells via the GLUT2 transporter, where it generates reactive oxygen species and induces oxidative damage.^53^, ^54^ Alloxan is commonly administered as alloxan monohydrate—owing to its solubility—through IP, IV, or SC routes.^55^

Despite its historical importance, alloxan is now used less frequently due to its inconsistent diabetogenic response and narrow therapeutic window. Hyperglycemia following alloxan administration is often transient, with spontaneous β‐cell regeneration leading to recovery within ~2 weeks in many species.^9^, ^56^ Moreover, alloxan frequently causes acute insulin release immediately after injection, precipitating potentially fatal hypoglycemia unless rapidly managed with glucose supplementation.^57^ In addition, alloxan has a narrow effective dose range; subtherapeutic doses result in incomplete β‐cell destruction and spontaneous recovery, whereas supratherapeutic doses are frequently lethal. A recent study reported that all rats treated with alloxan at doses ranging from 120 to 180 mg/kg fully recovered from hyperglycemia, whereas only animals receiving low‐dose STZ (40 mg/kg) exhibited reversible hyperglycemia; higher doses of STZ produced permanent insulin‐dependent diabetes. To achieve sustained hyperglycemia with alloxan, relatively high doses (100–200 mg/kg) are typically required, increasing the risk of systemic toxicity, especially affecting nontarget tissues.^9^, ^52^, ^58^, ^59^

The diabetogenic reliability of alloxan is strongly species dependent. In rodents such as hamsters, IP alloxan (50–250 mg/kg) often induces only temporary hyperglycemia, with full metabolic recovery due to robust β‐cell regeneration, as demonstrated in classic studies by Phares.^60^ In contrast, larger animals exhibit far greater susceptibility. Wang et al.^61^ showed that a single IV dose of 100 mg/kg produced persistent hyperglycemia lasting up to 1 year in New Zealand White rabbits, with consistent β‐cell destruction and secondary complications, including renal vascular lesions and hepatic lipid accumulation.

Alloxan has also been applied—though infrequently—in adult zebrafish. IP doses of 100–350 mg/kg trigger a brief, high‐amplitude hyperglycemic response, often requiring sucrose rescue to prevent fatal hypoglycemia; however, STZ produces more durable β‐cell ablation and is therefore preferred in teleost models.^62^

Mechanistically, alloxan acts primarily through hydroxyl‐radical–mediated oxidative stress rather than DNA alkylation, which contributes to its variability compared to STZ.^63^ Its marked chemical instability in aqueous solution mandates immediate preparation prior to dosing, further reducing its practical utility.^57^ For these reasons, alloxan accounts for only ~30% of chemical induction studies, whereas STZ is used in ~58% of cases,^9^ reflecting its superior reproducibility and long‐term metabolic stability.^9^

#### 2.1.3 Dithizone

Dithizone (diphenylthiocarbazone) is a sulfur‐containing zinc‐chelating agent that selectively targets pancreatic β cells owing to their high zinc content. After IV or IP administration—routes most frequently used in experimental studies—dithizone rapidly enters the pancreatic islets and forms zinc–dithizonate complexes within 2–5 min, leading to β‐cell necrosis and acute insulin deficiency.^64^, ^65^, ^66^

Although diabetogenic doses of dithizone do not cause acute systemic toxicity in rabbits, chronic progression of dithizone‐induced diabetes has been associated with parenchymal dystrophy and early fibrotic alterations in the liver, kidneys, lungs, and heart, largely attributable to sustained hyperglycemia and the toxic metabolic effects of zinc–chelator complexes.^67^ Dithizone‐induced diabetes was primarily used in earlier studies to investigate the physiological role of zinc in pancreatic β‐cell function, but its current use is limited due to toxicity concerns and the availability of more specific β‐cell–selective toxins.^27^, ^68^

#### 2.1.4 Gold thioglucose

Gold thioglucose (GTG), with the chemical formula AuSC_6_H_11_O_5_, is used experimentally to induce T2D through targeted damage to the hypothalamus. After IP injection, GTG selectively injures the ventromedial hypothalamus, leading to hyperphagia, progressive weight gain, and, ultimately, obesity. Over a period of 16–20 weeks, animals develop hallmark metabolic abnormalities of T2D, including hyperinsulinemia, insulin resistance, hyperglycemia, and dyslipidemia.

Unlike β‐cell toxins such as STZ, GTG induces diabetes indirectly by disrupting central appetite regulation rather than directly affecting pancreatic β cells. This characteristic makes GTG a valuable model for studying hypothalamic obesity and the neuroendocrine mechanisms underlying metabolic syndrome.^69^, ^70^, ^71^

#### 2.1.5 Other approaches

Other chemical approaches for diabetes induction, such as potassium xanthate, ferric nitrilotriacetate, STZ analogs, and monophosphoimidazolyl–dithiolacetamide, have been described but remain less commonly used. Notably, combined protocols (e.g., alloxan and STZ coadministration) have been applied in dogs and cats, where lower doses of each agent are given concurrently to induce diabetes while minimizing extrapancreatic toxicity.^72^ Similarly, monosodium glutamate (MSG) has been used to induce hypothalamic injury and obesity in neonatal models, which is followed by STZ administration to further induce metabolic dysfunction. These models primarily focus on metabolic syndrome rather than diabetes itself.^73^

#### 2.1.6 Summary of chemical models: Applications, advantages, and limitations

Chemical induction models enable rapid, cost‐effective, and controllable induction of hyperglycemia, making them particularly suitable for acute pharmacological testing, mechanistic studies of β‐cell loss, and evaluation of antidiabetic interventions. High‐dose STZ and alloxan primarily model insulin‐deficient T1D, whereas low‐dose or combined protocols (e.g., STZ with nicotinamide or HFD) better mimic late‐stage T2D characterized by partial β‐cell dysfunction and insulin resistance. Despite these advantages, substantial interanimal variability in postinduction fasting glucose and insulin levels remains a major limitation. This variability is influenced by strain sensitivity, age, sex, nutritional status, and inconsistencies in STZ preparation and administration; therefore, identical dosing protocols may not result in uniform β‐cell destruction, and metabolic outcomes should be interpreted as ranges rather than fixed values. Additional limitations include potential off‐target toxicity and limited representation of autoimmune or obesity‐driven pathophysiology unless specifically incorporated.

### 2.2 Dietary and metabolic models

Dietary models induce diabetes by creating overnutrition‐driven metabolic stress, primarily replicating T2D and metabolic syndrome, as diet alone rarely produces absolute insulin deficiency. The overarching goal is to mimic human dietary patterns that promote obesity, insulin resistance, and progressive glucose intolerance.

The most widely used approach is the HFD model, in which rodents—typically C57BL/6 mice or Wistar/Sprague–Dawley rats—receive an obesogenic diet containing 45%–60% kcal from fat for several weeks. This regimen reproduces key components of the Western dietary pattern^26^, ^74^ and, over 12–20 weeks, induces obesity, increased visceral adiposity, hepatic steatosis, hyperinsulinemia, and ultimately insulin resistance and glucose intolerance.^26^, ^75^ However, most rodents maintain near‐normal fasting glucose through compensatory hyperinsulinemia, making overt diabetes uncommon unless an additional β‐cell stressor is applied. The percentage and type of fat are critical: moderate HFDs (~45% kcal) more closely reflect human nutrition than extreme HFDs (60%–70% kcal).^75^, ^76^ Typical dietary compositions, feeding durations, and combination protocols used for induction of T2D‐like phenotypes are comparatively summarized in Table 3 to facilitate reproducibility and cross‐study comparison.

To promote overt diabetes, HFD is frequently combined with low‐dose STZ, which partially impairs β‐cell function and removes compensatory insulin responses. In these hybrid protocols, animals receive HFD (often supplemented with 10%–20% sugar) for 4–8 weeks, followed by STZ at 25–40 mg/kg.^26^, ^77^, ^78^ This results in obesity, insulin resistance, moderate β‐cell loss, and stable hyperglycemia, modeling late‐stage T2D.^26^, ^79^ For example, Wickramasinghe et al.^79^ showed that 4‐week HFD followed by 30–50 mg/kg STZ in Wistar rats induced marked dyslipidemia, elevated HbA1c, and dose‐dependent glycemic abnormalities. The severity of metabolic disturbances depends on the STZ dose: 50 mg/kg induced more pronounced glycemic and lipid abnormalities than 30 mg/kg.

Other diet‐based models include high‐sugar diets (fructose‐ or sucrose‐rich), cafeteria diets, and Western‐type diets high in fat and cholesterol. These rapidly induce insulin resistance, hepatic steatosis, and, over months, glucose intolerance and hyperglycemia.^80^ For instance, long‐term feeding of high‐energy diets has been shown to increase fasting glucose, HbA1c, and urinary glucose within ~30 weeks.^26^, ^81^, ^82^ Although these models effectively recapitulate components of metabolic syndrome, long exposure periods and strain‐dependent variability limit consistency.

Large‐animal dietary models, particularly in minipigs, provide a closer physiological match to human lipid and glucose metabolism. Xi et al.^83^ demonstrated that high‐fat/high‐sucrose feeding induced progressive obesity, insulin resistance, impaired glucose tolerance, and atherosclerotic lesions in minipigs, closely mimicking human cardiometabolic disease. Similarly, Zhao et al.^84^ showed that Bama minipigs fed an HFD for 3 months and then challenged with STZ developed stable T2D, confirming their utility for modeling diet‐related metabolic dysfunction. These models offer superior translational relevance due to similarities in gastrointestinal physiology, lipid handling, and β‐cell responsiveness compared to rodents.

Diet‐induced metabolic dysfunction has also been replicated in nonhuman primates. In rhesus macaques, a chronic high‐fat or high‐fructose diet leads to central obesity, insulin resistance, dyslipidemia, inflammation, and progression toward overt T2D in a subset of animals.^85^ These primates demonstrate elevated fasting glucose and insulin, impaired glucose tolerance, and early β‐cell failure, providing one of the most human‐relevant metabolic platforms. Likewise, cynomolgus monkeys fed long‐term HFD exhibit gradual development of obesity and T2D, paralleling human metabolic syndrome.

Golden Syrian hamsters represent another useful large‐rodent model. High‐fructose diets (e.g., 60% fructose) cause obesity, hypertriglyceridemia, and insulin resistance, as shown by Kasim‐Karakas et al.,^86^ who observed rapid weight gain, reduced glucose disappearance rates, and significant triglyceride elevation. Similar metabolic impairments occur with HFD, including marked hepatic lipid accumulation and insulin resistance.^87^

Although chronic or binge alcohol intake can impair glucose homeostasis and insulin signaling, alcohol‐based liquid diets are not considered reliable diabetes‐inducing models. As reviewed by Kim et al.,^88^ alcohol consumption produces highly variable and nonspecific metabolic effects, including hepatic injury and pancreatitis but lacks the reproducibility and β‐cell–targeted cytotoxicity required for validated T1D or T2D models. Therefore, alcohol‐containing diets are better suited for studying alcohol‐related metabolic disturbances rather than diabetes induction.

Dietary and metabolic models are best suited for studying obesity‐driven insulin resistance, dyslipidemia, hepatic steatosis, and early metabolic dysfunction preceding overt diabetes. High‐fat, high‐sugar, and Western‐type diets closely reflect human lifestyle‐related T2D risk factors and are valuable for evaluating nutritional or lifestyle interventions. However, these models typically exhibit slow and variable onset of hyperglycemia, strong strain dependence, and limited β‐cell failure, often requiring combination with chemical or genetic stressors to induce stable diabetes. Comparative details for these models are summarized in Tables 2 and 3.

**TABLE 2:** Common type 1 diabetes (T1D) models in laboratory animals.

Model (induction dosage and routes of administration)		Species/strain	Onset (time)	Key features	Advantages	Limitations	References
Chemicals	STZ (100–200 mg/kg, IP/IV)	Mouse, minipigs (Goettingen /Wuzhishan), cynomolgus	Rapid (days)	Near‐complete β‐cell necrosis; acute insulin deficiency; sustained hyperglycemia	Simple, fast, and reproducible; applicable across species	Requires precise dosing; risk of initial hypoglycemia; renal/hepatic toxicity; mutagenic	[33, 34, 35, 43, 44, 45, 72]
	Alloxan (100–200 mg/kg, IP/IV)	Rat (Wistar), mouse, rabbit, zebrafish, hamster	Acute (hours–days)	β‐cell destruction via ROS; induces hyperglycemia	Inexpensive; historically established	High mortality due to insulin shock; transient diabetes due to β‐cell regeneration; variable response; nephrotoxic	[9, 52, 58, 59, 61, 72]
	MLD‐STZ (20–40 mg/kg/day, IP)	Mouse (C57BL/6), rat	Slow (weeks)	Gradual immune‐mediated β‐cell destruction; mimics autoimmune insulitis	Models chronic onset and autoimmunity; more physiological progression	Requires multiple injections; strain‐ and sex‐dependent variability; partial nonimmune toxicity	[36, 37, 38]
	Cyclophosphamide (200 mg/kg, IP) + STZ (20–40 mg/kg, IP)	Mouse (NOD, C57BL/6), rat	Weeks	Immune‐triggered β‐cell destruction; accelerated autoimmune response	Synchronizes T1D onset; efficient for autoimmune studies	Cyclophosphamide toxicity (e.g., bladder); broad immunosuppression; ethical concerns	[26]
	Dithizone (40–60 mg/kg, IV)	Cat, mouse, rabbit, hamster	Days	Selectively destroys insulin‐storing β‐cell granules	Historical use; specific β‐cell staining	Rarely used; limited metabolic data; off‐target toxicity; largely replaced by STZ/alloxan	[67, 68]
Surgical	Total/subtotal Pancreatectomy (50%–90% removing, Surgical)	Rat, dog, Goettingen minipigs	Immediate (postsurgery)	Surgical removal (~90%) of pancreas; total insulin dependence	No chemical toxicity; precise control over β‐cell mass	Invasive; requires surgical expertise; high mortality and malabsorption	[45, 89, 90, 91, 92, 93]
Viral	EMCV infection	Mouse, rat	Days–weeks	Virus‐induced pancreatitis and β‐cell necrosis	Mimics environmental triggers; immune component involved	Strain‐specific susceptibility; variable onset; biosafety issues	[26, 94, 95]
	Coxsackievirus B infection	Mouse (e.g., CVB4), rat	Days–weeks	Viral islet infection; autoimmune β‐cell targeting	Models infection‐induced T1D; relevant for studying immune activation	Low reproducibility; host‐specific; limited to virus‐susceptible strains	[96, 97, 98, 99, 100]
Genetic models	NOD mouse (spontaneous)	NOD/ShiLtJ	~12–30 weeks	Spontaneous autoimmune insulitis; polygenic; ~60%–80% incidence in females	High resemblance to human T1D; no induction needed	Long latency; sex bias; costly maintenance; limited translational predictability	[101, 102]
	BB rat (spontaneous)	BioBreeding (Wistar‐derived)	~8–16 weeks	Spontaneous autoimmune β‐cell loss; lymphopenia	High penetrance; polygenic T1D model	Lymphopenia uncommon in human T1D; limited to rats; high maintenance cost	[103, 104, 105]
	LEW.1AR1‐iddm rat	LEW (IDDM)	~8–12 weeks	MHC‐linked autoimmune insulitis; polygenic model	Defined genetic background; autoimmunity‐relevant	Specialized strain; moderate incidence; limited availability	[53, 94, 106]
	LETL rat (spontaneous)	Long‐Evans Tokushima Lean	~20–28 weeks (~5–6 months)	Autoimmune insulitis and β‐cell destruction; classic T1D histopathology	Closely mimics human T1D pathology	Low penetrance (~20%); slow progression; expensive upkeep	[26, 107]

Abbreviations: EMCV, encephalomyocarditis virus; IP, intraperitoneal; IV, intravenous; MLD, multi‐low dose; NOD, nonobese diabetic mouse; ROS, reactive oxygen species; STZ, streptozotocin.

**TABLE 3:** Common type 2 diabetes (T2D) models in laboratory animals.

Model (induction dosage and routes of administration)		Species/strain	Onset (time)	Key features	Advantages	Limitations	References
Dietary	HFD (60%–70% kcal, oral)	Mouse (C57BL/6J, etc.), rat	Weeks–months (≥12–20 week)	Diet‐induced obesity, insulin resistance, hyperinsulinemia	Physiologically relevant; mimics Western‐style diet and obesity	Slow onset; often only impaired glucose tolerance without frank diabetes; variability by strain/sex	[26, 74, 75, 76]
	High‐sugar/fructose diet (10% fructose water, oral)	Rat, mouse	Months (chronic)	Excess sugar consumption leads to insulin resistance, fatty liver, mild hyperglycemia	Models effects of dietary sugar; often combined with HFD	Alone usually insufficient for established diabetes; requires long duration; effects subtle	[26, 80, 81, 82]
Dietary + chemicals	HFD (45%–60% kcal, oral) + modest‐dose STZ (25–50 mg/kg, IP)	Rat (Wistar), mouse (C57BL/6)	~4–12 weeks (postdiet)	Combines diet‐induced IR with partial β‐cell injury; sustained hyperglycemia	Reproducible T2D‐like state; moderate obesity; shorter induction time	Critical STZ dosing; partial β‐cell loss may vary; still partly artificial compared to pure polygenic T2D	[26, 77, 78, 79]
	HFD/HCFD (oral) + STZ (90 mg/kg, IP)	Minipigs (Bama)	After 3 months	Stable T2D phenotype in HFD + STZ and HCFD+STZ groups	Relevance to human pathophysiology	Requirement for long‐term exposure, variability in glycemic outcomes	[84]
Chemicals	STZ (40–125 mg/kg, IP/IV) + NA (67–230 mg/kg, IP/IV)	Rat, mouse, micropigs	Weeks (chronic)	STZ‐induced partial β‐cell destruction protected by NA; moderate, stable hyperglycemia	Nonobese T2D model; retains some β‐cell function (milder diabetes)	Limited insulin resistance component; simpler pathology; must optimize NA dose	[39, 40, 41, 42, 46]
Genetic models	ob/ob mouse (Lep^ob^)	Mouse (C57BL/6J‐ob/ob)	4–6 weeks	Monogenic leptin‐deficient obesity; hyperphagia, severe obesity, IR; moderate hyperglycemia	Genetically stable; easy breeding; pronounced obesity/IR	Leptin deficiency is rare in humans; mice often compensate with high insulin (diabetes can be mild or diet dependent)	[108, 109, 110]
	db/db mouse (Lepr^db^)	Mouse (C57BL/Ks or B6–db/db)	~8–10 weeks (males)	Monogenic leptin‐receptor mutation; extreme obesity, IR, marked hyperglycemia	Consistent, severe diabetic phenotype (especially males)	Same leptin‐pathway issue as ob/ob; mice are severely obese (differences from human T2D); background strain affects severity	[109, 111, 112, 113, 114]
	Zucker diabetic fatty (ZDF) rat	Rat (fa/fa, Zucker strain)	~8–16 weeks (males)	Leptin‐receptor mutation (fa/fa); obesity, IR; β‐cell failure leads to hyperglycemia	Well‐characterized male rats reliably develop diabetes	Requires specific Purina diet for full phenotype; females often do not develop diabetes; model needs careful husbandry	[115, 116, 117, 118]
	Goto‐Kakizaki (GK) rat	Rat (Wistar‐derived GK)	~12 weeks	Polygenic; β‐cell dysfunction, moderate fasting hyperglycemia, insulin resistance	Genetic, nonobese T2D model; useful for chronic studies and complications	Mild‐to‐moderate hyperglycemia (e.g., 180–250 mg/dL); lack of obesity means different pathology than typical human T2D	[119, 120, 121, 122, 123]
	Long‐Evans Tokushima Fatty (OLETF)	Rat (OLETF)	20–30 weeks	Mild obesity, IR; late‐onset progressive hyperglycemia	Models adult‐onset T2D; pathophysiology similar to human adult T2D	Slow development (requires long‐term studies); relatively moderate phenotype; strain availability limited	[122, 124]
	KK‐Ay	Mouse (KK background with Ay)	Early (weeks)	Aggressive polygenic obesity and diabetes; hyperphagia, severe obesity, IR	Early‐onset diabetes with intact leptin signaling; models genetic/obese T2D	Extremely obese and yellow coat may complicate some studies; aggressive phenotype can reduce generalizability	[125, 126]
Hormonal	Glucocorticoid (dexamethasone, 0.1–1 mg/kg/day, IP/oral)	Rat, mouse	Days (chronic)	Systemic insulin resistance; hyperglycemia, dyslipidemia (Cushing‐like syndrome)	Models steroid‐induced diabetes; highlights mechanisms of insulin resistance	Β cells largely intact (not T1D); general catabolic and immunosuppressive effects; requires prolonged hormone exposure	[127, 128, 129, 130, 131, 132, 133, 134]

Abbreviations: HCFD, high‐carbohydrate, high‐fat diet; HFD, high‐fat diet; IP, intraperitoneal; IR, insulin resistance; IV, intravenous; STZ, streptozotocin.

### 2.3 Surgical models

Surgical models induce diabetes by physically reducing β‐cell mass and are primarily employed to study β‐cell regeneration, metabolic compensation, and islet plasticity.^53^ Although historically more common in large‐animal research, these procedures have also been applied in rodents.

#### 2.3.1 Total pancreatectomy

Total pancreatectomy (TP)—the complete removal of the pancreas—results in immediate and absolute insulin deficiency, producing a diabetic state that closely mirrors late‐stage T1D.^89^, ^90^ This approach has been used in dogs, pigs, nonhuman primates, and occasionally rodents. However, TP is technically demanding, requires advanced surgical expertise, causes complete exocrine insufficiency, and carries significant ethical and perioperative challenges.^135^, ^136^ Postoperative care typically includes intensive insulin therapy and pancreatic enzyme replacement, and mortality rates are high in small animals such as mice.^26^, ^91^, ^137^ For these reasons, TP is now seldom used in rodent studies. In larger species, such as Göttingen minipigs, TP reliably eliminates endogenous insulin and C‐peptide but still demands substantial postoperative support and resources, offering no major practical advantage over STZ‐induced diabetes despite its physiological fidelity.^45^

#### 2.3.2 Partial pancreatectomy

Partial pancreatectomy (PPx) is more widely used due to lower surgical burden and greater survival rates. Removing 50%–90% of pancreatic tissue produces proportional β‐cell loss: limited resections yield mild glucose intolerance, whereas extensive (e.g., 90%) resections produce sustained hyperglycemia and moderate‐to‐severe diabetes.^92^ The classic 90% PPx rat model was originally developed to circumvent chemical agent toxicity,^26^ and Bonner‐Weir et al.^93^ demonstrated that 90% PPx produces stable hyperglycemia lasting several weeks, making it a valuable platform for studying β‐cell adaptation and regeneration.

PPx is also frequently combined with secondary interventions to enhance model versatility. For example, PPx followed by isoproterenol treatment or unilateral nephrectomy has been used to model diabetic cardiomyopathy and nephropathy.^138^ Likewise, PPx combined with low‐dose alloxan or STZ generates reproducible moderate diabetes with reduced systemic toxicity compared to full‐dose chemical induction.^139^ More recently, PPx has become a central tool for regeneration research: after substantial pancreatic resection, islet regrowth, neogenesis, and compensatory proliferation can be examined under various molecular or pharmacological stimuli.^26^, ^140^

In large‐animal and primate studies, total and subtotal pancreatectomy continue to serve as highly controlled models of insulin deficiency. For instance, TP in cynomolgus and rhesus monkeys produces stable, insulin‐dependent diabetes (fasting glucose >400 mg/dL) but necessitates extensive postoperative management, including enzyme supplementation. To minimize surgical morbidity, subtotal pancreatectomy (70%–80% tissue removal) is often preferred. In one study, Fathi et al.^141^ performed ~50% pancreatectomy followed by low‐dose STZ (30–60 mg/kg), achieving sustained diabetes with minimal hypoglycemia—an approach that effectively balances surgical precision with reduced chemical toxicity.

#### 2.3.3 Summary of surgical models: Applications, advantages, and limitations

Surgical models provide precise and controlled reduction in β‐cell mass, making them valuable for studying β‐cell regeneration, metabolic compensation, and islet plasticity. Total pancreatectomy closely mimics absolute insulin deficiency, whereas partial pancreatectomy enables graded metabolic impairment. Despite their physiological relevance, surgical models have important limitations. These include invasiveness, technical complexity, and postoperative morbidity, as well as confounding effects of exocrine insufficiency, which collectively restrict their routine use, particularly in small animals.

### 2.4 Genetically and spontaneously diabetic models

A wide range of rodent strains develop diabetes spontaneously due to inherited mutations. These models can be either monogenic, involving defects in a single gene, or polygenic, reflecting the interaction of multiple genetic risk factors. They often replicate specific diabetes subtypes—such as autoimmune T1D or obesity‐linked T2D—and exhibit well‐characterized and reproducible phenotypes. Genetically and spontaneously diabetic models provide a powerful platform for investigating the natural pathophysiology of diabetes in the absence of chemical agents or invasive interventions, making them particularly valuable for longitudinal studies and mechanistic research.^72^

#### 2.4.1 Monogenic and polygenic models of T2D

- ob/ob mouse (Lep^ob^): This mouse harbors a mutation in the leptin gene, leading to severe obesity, hyperphagia, hyperinsulinemia, and mild‐to‐moderate hyperglycemia.^108^ ob/ob mice become profoundly obese by 4–6 weeks of age. Although they develop insulin resistance, most compensate with elevated insulin levels, and established diabetes may be absent depending on the genetic background.^109^ This model is commonly used to study obesity and insulin resistance, though its diabetic phenotype may be milder compared to other models.^110^
- db/db mouse (Lepr^db^): These mice lack the leptin receptor.^111^ Like ob/ob mice, they develop obesity and insulin resistance but exhibit more severe hyperglycemia.^109^, ^112^ On the C57BL/Ks background, male db/db mice develop extreme hyperglycemia (>300 mg/dL) by 8–10 weeks of age, accompanied by progressive β‐cell failure.^113^, ^114^ In contrast, the phenotype is milder on the C57BL/6 background. These mice model T2D with both insulin resistance and relative insulin deficiency, though their extreme obesity limits translational relevance to lean T2D in humans.^109^
- Zucker diabetic fatty (ZDF) rat: The ZDF rat carries a fa/fa mutation in the leptin receptor gene, similar to the db/db mouse.^115^ On the Zucker genetic background, homozygous animals develop obesity, insulin resistance, and overt hyperglycemia by adulthood. Male ZDF rats typically exhibit hyperinsulinemia initially, followed by declining insulin levels and diabetes onset around 12 weeks of age. This model is widely recognized for studying T2D in rats.^116^ A related strain, the ZDSD rat, is an outbred model that develops diabetes without extreme leptin pathway mutations, making it more representative of polygenic human T2D.^117^, ^118^
- Goto‐Kakizaki (GK) rat: The GK rat is a nonobese, polygenic model of T2D, developed by selective inbreeding of glucose‐intolerant Wistar rats.^119^, ^142^ These animals develop moderate fasting hyperglycemia, insulin resistance, and impaired insulin secretion by ~4 weeks of age.^120^, ^121^, ^122^ GK rats are useful for studying T2D in lean individuals and its complications, such as microvascular damage, although they exhibit only moderate hyperglycemia.^123^
- Otsuka Long‐Evans Tokushima Fatty (OLETF) rat: The Otsuka Long‐Evans Tokushima fatty rat spontaneously develops mild‐to‐moderate T2D and obesity. Disease onset is adult and sex dependent. Its pathogenesis involves a mutation in the cholecystokinin‐A receptor.^122^, ^124^
- KK/Ay mouse: This polygenic T2D model is created by introducing the dominant yellow agouti (Ay) mutation into the diabetic‐prone KK mouse background. KK/Ay mice exhibit early‐onset hyperphagia, obesity, hyperinsulinemia, and persistent hyperglycemia. Unlike leptin‐deficient models, KK/Ay mice retain functional leptin signaling, making them valuable for studying obesity‐driven T2D and its complications, such as bone diseases.^125^, ^126^
- Monogenic β‐cell models: Genetically engineered mice lacking key genes involved in β‐cell development or insulin signaling (e.g., Ins1/Ins2 double knockouts, Pdx1 knockouts, insulin receptor knockouts) serve as targeted models of β‐cell failure or insulin resistance.^143^, ^144^, ^145^ Although not typically used for pharmacological testing, they help elucidate specific pathophysiological pathways.

#### 2.4.2 Spontaneous autoimmune models of T1D

- Nonobese diabetic (NOD) mouse: The NOD mouse is the prototypical model of autoimmune T1D. These inbred mice spontaneously develop insulitis and T‐cell–mediated β‐cell destruction, resulting in diabetes in 60%–80% of females and 20%–30% of males.^101^ The model recapitulates many aspects of human T1D, including major histocompatibility complex (MHC)‐linked genetic susceptibility,^146^ insulitis, T‐cell infiltration, and secondary complications like nephropathy and neuropathy. NOD mice are essential for studying autoimmune mechanisms and immunotherapy. Limitations include incomplete overlap of human and murine autoantigens and poor translational efficacy of some successful treatments.^101^ Additionally, NOD mice are immunologically fragile and costly to maintain.^102^
- BioBreeding (BB) rat: Derived from Wistar rats, BB rats develop autoimmune diabetes spontaneously in 60%–90% of animals between 50 and 90 days of age. They exhibit lymphopenia, particularly of CD4^+^ T cells, contributing to immune dysregulation. The model is characterized by insulitis, progressive β‐cell destruction, and a high incidence of diabetes. Genetic studies have identified key mutations responsible for their autoimmune phenotype.^103^, ^104^, ^105^
- LEW.1AR1‐iddm and Komeda (KDP) rats: These less commonly used rat models harbor distinct MHC/immune‐related mutations and spontaneously develop autoimmune T1D. LEW.1AR1‐iddm rats develop lymphocytic insulitis, ^106^, ^107^ whereas Komeda rats (a Lewis substrain) display similar patterns of β‐cell destruction.^53^ Although less widely used than NOD or BB rats, both models share the key feature of T‐cell–mediated autoimmunity against pancreatic islets.^53^
- Long‐Evans Tokushima Lean (LETL) rat: This was the first rat model of spontaneous autoimmune T1D. About 20% of LETL rats develop diabetes by 5–6 months of age, exhibiting classical symptoms and histopathological features such as insulitis and β‐cell destruction.^26^ Two recessive diabetes loci linked to the RT1u MHC haplotype contribute to disease susceptibility.^107^ Although historically important, low penetrance and high maintenance costs limit its widespread use.

Dogs represent an important large‐animal model for T1D, closely mimicking human diabetes. Unlike rodents, dogs naturally develop insulin‐dependent diabetes, exhibiting progressive β‐cell loss and chronic complications. Early studies by Engerman and Kramer demonstrated that both spontaneous and chemically induced diabetic dogs show similar metabolic and pathological features to human diabetes.^147^ More recently, Adin and Gilor emphasized the value of diabetic dogs for translational islet transplantation research, highlighting their similarities in pancreatic anatomy, immune response, and glycemic dynamics with humans.^148^ These features position dogs as a complementary large‐animal system, ideal for evaluating long‐term diabetes progression and advanced therapeutic interventions.

Genetically modified diabetic hamsters have also emerged as a novel model. A CRISPR knockout of the insulin receptor substrate‐2 gene (IRS2) in golden hamsters produced a nonobese T2D model, exhibiting hyperglycemia and β‐cell hypoplasia without obesity, which mirrors certain human T2D subtypes.^149^

Lastly, zebrafish models have been genetically engineered to study diabetes. The most well‐characterized is the *pdx1* mutant: homozygous *pdx1* ^−/−^ zebrafish have markedly reduced β‐cell mass, low insulin, and high glucose. These fish display key diabetic features (elevated fasting glucose and impaired islets) and respond to antidiabetic drugs.^150^ Other mutants include insulin gene knockouts and hyperglycemic lines (e.g., *insa*, *gck*, and *glut2* mutants) and, recently, CRISPR‐engineered strains. A *pdx1* knockout also leads to diabetic nephropathy‐like renal changes.^151^

#### 2.4.3 Summary of genetic models: Applications, advantages, and limitations

Genetic and spontaneous models recapitulate the natural onset and progression of diabetes driven by defined monogenic defects or polygenic susceptibility. These models are particularly valuable for investigating long‐term disease mechanisms, gene–environment interactions, and chronic complications. Obese genetic models (e.g., ZDF, db/db) are well suited for studying insulin resistance and β‐cell failure in T2D, whereas nonobese models (e.g., GK rats) better represent lean T2D phenotypes. Spontaneous autoimmune models closely mirror the immunopathogenesis of human T1D and are indispensable for studying immune‐mediated β‐cell destruction. However, limitations include extreme obesity or developmental abnormalities in some models, immune dysfunction, sex‐ and strain‐dependent variability, delayed or incomplete disease penetrance, and high maintenance and breeding costs. Consequently, careful model selection based on the specific research objective—autoimmunity versus insulin resistance—is essential for maximizing translational relevance (Tables 2 and 3).

### 2.5 Immune‐mediated and antigen‐induced models

Beyond spontaneous models, diabetes can also be experimentally induced by modulating the immune system to mimic the autoimmune destruction of pancreatic β‐cells—hallmark of T1D.

- MLD‐STZ: Administering STZ in repeated low doses (e.g., 40 mg/kg for 5 consecutive days) not only causes gradual β‐cell destruction but also initiates an inflammatory response characterized by T‐cell infiltration and cytokine release in pancreatic islets.^26^, ^152^ This model is thus considered an immune‐mediated approximation of T1D and is frequently used in nongenetic backgrounds to evaluate immunopathology and immunomodulatory therapies.
- Cyclophosphamide‐augmented NOD: In NOD mice, cyclophosphamide accelerates disease onset by selectively depleting regulatory T cells, thereby enhancing autoreactive T‐cell activity. This approach is commonly used to synchronize disease progression and amplify autoimmune responses for experimental consistency.^153^
- Adoptive transfer models: Transferring diabetogenic T cells or immune serum from diabetic donors into immunodeficient recipients (e.g., NOD‐SCID) can reproduce autoimmune diabetes.^154^, ^155^ Although not a routine model, this strategy is invaluable for confirming the pathogenic role of specific immune cell populations.
- Antigen‐based and vaccinogenic models: Immunization with pancreatic autoantigens (such as insulin or GAD65 peptides) in conjunction with adjuvants can break immune tolerance and elicit T1D‐like pathology. For instance, GAD‐based immunization in susceptible strains induces insulitis and β‐cell loss. However, these models are complex, require careful optimization, and often show poor reproducibility.^156^, ^157^, ^158^
- Checkpoint inhibitor models: With the advent of cancer immunotherapy, agents such as anti–PD‐1 or anti–CTLA‐4 antibodies have been employed in experimental models to induce T1D by unleashing autoreactive T cells. These models simulate iatrogenic diabetes observed in patients undergoing immune checkpoint blockade, offering insight into immune tolerance breakdown.^99^, ^159^, ^160^

Immune‐mediated and vaccinogenic models replicate key aspects of autoimmune β‐cell destruction and provide a physiologically relevant immune context, making them particularly valuable for investigating the pathogenesis of T1D, immune tolerance breakdown, and the evaluation of immunotherapeutic strategies. These models induce diabetes through diverse mechanisms, including gradual β‐cell damage, T‐cell activation, and loss of immune tolerance, offering advantages over direct chemical or genetic induction. However, their experimental complexity, strong dependence on genetic background, and limited reproducibility restrict their applicability for large‐scale or standardized drug screening, rendering them more suitable for mechanistic and hypothesis‐driven studies rather than routine experimental use. Standardization of dosing regimens, animal age and sex, genetic background, housing conditions, and immunization protocols may improve interstudy reproducibility and translational consistency of these immune‐mediated models.

### 2.6 Viral models

Viral infections have long been implicated as triggers of T1D, and certain viruses can induce diabetes in susceptible hosts.^100^ The most‐studied diabetogenic virus is encephalomyocarditis virus (EMCV), an enterovirus in the Picornaviridae family.^26^, ^95^ Experimental studies typically employ naturally occurring strains, particularly the diabetogenic D variant, which exhibits enhanced tropism for pancreatic β cells. In genetically susceptible mouse strains, EMCV‐D induces pancreatitis and β‐cell necrosis, leading to hyperglycemia.^94^, ^161^ Similarly, Coxsackievirus B (CVB) strains used in experimental models are generally wild‐type or mouse‐adapted isolates rather than genetically engineered variants. These viruses can infect pancreatic islets and, under specific immunogenetic conditions, precipitate autoimmune diabetes. In contrast, certain models such as the rat insulin promoter (RIP)‐lymphocytic choriomeningitis virus (LCMV) system rely on transgenic mice expressing viral antigens specifically in pancreatic β cells under the control of the RIP. In this model, the viral genome itself is not genetically modified; rather, the host mouse is engineered to express LCMV‐derived antigens in β cells, enabling the study of antigen‐specific immune‐mediated β‐cell destruction.^94^, ^96^ Rotaviruses and LCMV have also been investigated using primarily natural viral strains, although disease induction depends strongly on host susceptibility and experimental context.^97^, ^98^, ^162^, ^163^, ^164^

However, viral models are not widely used in standard diabetes research due to the complexity of infection and requirement for susceptible genetics. They are primarily tools to study virus–host interactions and T1D triggers. Recent reviews of virus‐induced diabetes emphasize host susceptibility factors (e.g., presence of viral receptors on β‐cells) in determining disease outcome.^161^ For example, Coxsackie B virus causes overt diabetes only if mice have ongoing insulitis (as in NOD); in diabetes‐resistant strains it may have no effect or even transiently protect against autoimmunity.^161^, ^165^ Thus, although viruses are a recognized environmental factor, they are beyond the scope of routine model induction and are mainly of research interest.

Viral models are primarily research tools for studying environmental triggers of autoimmune diabetes and virus–host interactions. Although they offer insight into infection‐induced β‐cell damage and immune activation, their dependence on host susceptibility and experimental complexity limits their routine application in diabetes modeling.

### 2.7 Hormonal models

Hormone‐based models of insulin resistance are relatively uncommon and are primarily employed in mechanistic studies rather than standard protocols for diabetes induction. These models focus on counter‐regulatory hormones such as growth hormone (GH), glucagon, corticosteroids, and sex steroids, which antagonize insulin's hypoglycemic effects by promoting hepatic glucose production and impairing peripheral glucose uptake.^127^, ^166^

For example, transgenic mice overexpressing bovine GH exhibit peripheral insulin resistance but do not develop overt diabetes unless challenged with a HFD. This highlights the context‐dependent effects of GH on glucose metabolism.^167^ Similarly, glucagon infusion acutely increases blood glucose levels by hepatic gluconeogenesis. However, despite evidence of impaired glucose tolerance following prolonged infusion in rodents, glucagon is not suitable as a chronic model of T2D.^168^, ^169^ Estrogen deficiency induced by ovariectomy is frequently combined with an HFD to simulate postmenopausal insulin resistance, given the well‐established insulin‐sensitizing effects of estrogen. Estrogen replacement therapy has been shown to reverse metabolic impairments, including glucose intolerance and hepatic steatosis, in ovariectomized (OVX) rodent models.^170^, ^171^ Among these hormonal interventions, glucocorticoid (GC) excess is the most extensively studied. Chronic administration of synthetic GCs such as dexamethasone or prednisolone induces a Cushingoid metabolic phenotype characterized by insulin resistance, hyperglycemia, dyslipidemia, and hypertension in rodents.^127^, ^128^ Dexamethasone disrupts insulin signaling in skeletal muscle and adipose tissue while enhancing hepatic gluconeogenesis through upregulation of key gluconeogenic genes such as *PEPCK* and *G6Pase*.^129^, ^130^, ^131^ The resulting phenotype is marked by elevated fasting glucose levels and impaired glucose tolerance, typically without significant β‐cell dysfunction. This model is widely used to investigate the pathophysiology of insulin resistance and to evaluate insulin‐sensitizing agents due to its rapid onset and ease of induction, whether via injection or GC pellet implantation. However, it may not consistently induce hyperglycemia unless animals are obese or genetically predisposed, and systemic confounding factors such as catabolism and stress‐related responses must be carefully considered.^132^, ^133^, ^134^

Hormonal models primarily mimic insulin resistance induced by counter‐regulatory hormones and are useful for mechanistic studies of steroid‐ or stress‐related metabolic dysfunction. However, because β‐cell mass is largely preserved, these models do not replicate the full spectrum of T1D or T2D and are best suited for targeted investigations of insulin signaling impairment.

## 3 COMPARATIVE TABLES

To provide a comprehensive overview of commonly employed diabetic models, Table 2 summarizes representative methods for the induction of T1D‐like phenotypes, whereas Table 3 presents models used to induce T2D‐like conditions. Each model is categorized based on its induction method, commonly used species or strains, time of onset and disease severity, principal pathological and metabolic features, and its advantages, limitations, and key references.

## 4 MECHANISMS, COMPARISONS, AND LIMITATIONS OF DIABETES INDUCTION MODELS

Rather than reiterating model‐specific advantages and limitations, this section provides a comparative, mechanism‐oriented synthesis of diabetes induction strategies, highlighting how distinct pathophysiological pathways determine their experimental applications and constraints.

- β‐Cell destruction models in T1D: Chemical agents such as STZ and alloxan, or surgical approaches like total pancreatectomy, are used to induce insulin‐dependent diabetes by directly destroying pancreatic β cells. These models lead to severe insulin deficiency and are widely employed for mechanistic studies of absolute insulin deficiency, insulin replacement strategies, and the development of complications under sustained hyperglycemia. However, these methods bypass the autoimmune pathogenesis characteristic of human T1D, limiting their utility in immunological research. Moreover, partial recovery of β‐cell function, particularly with alloxan, may occur, and disease onset is typically abrupt. Graded administration of STZ, such as single low‐dose protocols, can model partial β‐cell dysfunction, offering an experimental platform for studying early or subclinical diabetes.^26^
- Autoimmune models (T1D): Models such as NOD mice, BB rats, and MLD‐STZ protocols replicate immune‐mediated β‐cell destruction characterized by both CD4^+^ T‐helper (predominantly Th1) and CD8^+^ cytotoxic T‐cell infiltration of pancreatic islets, accompanied by macrophage recruitment and pro‐inflammatory cytokine production (e.g., interferon γ [IFN‐γ], tumor necrosis factor α [TNF‐α], and interleukin 1β [IL‐1β]).^172^, ^173^, ^174^ These responses result in progressive insulitis and β‐cell apoptosis. Compared to toxin‐induced models, autoimmune models exhibit slower and more heterogeneous disease onset and are particularly valuable for studying immune tolerance breakdown, antigen presentation, and autoreactive T‐cell expansion. Major autoantigens implicated in these models include insulin, glutamic acid decarboxylase 65 (GAD65), IA‐2, and ZnT8, several of which are also recognized targets in human T1D‐associated autoimmunity.^175^ Compared to human T1D, these models reproduce several key features, including T‐cell–mediated β‐cell destruction and the presence of circulating autoantibodies. However, important differences remain. Human T1D typically exhibits a predominance of CD8^+^ T cells within islets and a more focal, heterogeneous insulitis pattern, whereas NOD mice often display peri‐insular and more organized lymphocytic infiltration.^176^ Moreover, differences in MHC genetics (e.g., murine H‐2 versus human leukocyte antigen [HLA] haplotypes) and immune regulation limit direct translational extrapolation.^101^, ^177^
- Insulin resistance models (T2D): Diet‐induced obesity models, including HFD and cafeteria diet regimens, primarily induce peripheral insulin resistance. Initially, β‐cell compensation through hyperinsulinemia maintains near‐normal glycemic levels; however, over time, this compensation fails, resulting in glucose intolerance. These models are often considered prediabetic and typically progress to overt diabetes only when additional stressors (genetic or chemical) are introduced. Although these models closely resemble lifestyle‐related T2D pathogenesis—characterized by obesity, insulin resistance, and dyslipidemia—they generally require extended feeding durations.^75^, ^178^, ^179^, ^180^
- Combined pathology models (T2D): Models that incorporate both β‐cell dysfunction and insulin resistance—such as NA + STZ, HFD + STZ, and genetically obese rodents (e.g., ob/ob, db/db mice)—more accurately simulate the multifactorial nature of human T2D. For example, STZ + NA rats retain some endogenous insulin secretion and develop many typical T2D complications, including nephropathy and dysregulation of the Nrf2 pathway.^39^, ^41^ ob/ob and db/db mice present with obesity‐induced insulin resistance followed by progressive β‐cell failure,^181^ making them particularly suitable for studying the transition from insulin resistance to insulin deficiency.
- Hormone‐induced models: Models based on glucocorticoid excess mimic stress‐related or iatrogenic diabetes (e.g., Cushing's syndrome). These animals exhibit pronounced insulin resistance in hepatic and muscular tissues, dyslipidemia, and moderate hyperglycemia. They are valuable for elucidating the mechanisms underlying steroid‐induced insulin resistance, such as impaired insulin signaling and increased gluconeogenesis.^130^ However, as β‐cell mass remains largely intact, these models are not appropriate for studying T1D pathophysiology.
- Genetic models: Rodents carrying single‐gene mutations (e.g., affecting leptin, insulin, or the insulin receptor) develop diabetes due to well‐defined molecular mechanisms. In these models, diabetes often arises from developmental abnormalities—for example, leptin deficiency during growth leads to excessive fat accumulation. These models provide powerful tools for investigating gene‐specific functions (e.g., leptin signaling in metabolism) but may not capture the multifactorial and heterogeneous nature of human diabetes.

Chemical and surgical models are distinguished by their ability to induce rapid and experimentally controlled hyperglycemia; however, this strength is offset by their limited representation of disease etiology. They are well suited for acute pharmacological studies. In contrast, dietary and genetic models more closely resemble human metabolic syndrome and reveal complex, chronic metabolic disturbances, though they typically progress more slowly and with greater interanimal variability. Autoimmune models are essential for understanding T1D pathogenesis but face challenges related to species‐specific immune responses. Collectively, no single experimental model fully recapitulates the complexity of human T1D or T2D. Each approach captures distinct disease components—ranging from β‐cell loss and insulin resistance to immune dysregulation—while omitting others. Accordingly, model selection should be driven by the specific biological process under investigation rather than by disease labels alone.

## 5 RECENT ADVANCES AND CONSIDERATIONS

Recent advancements in diabetes modeling have been driven by the rapid evolution of precision genome‐editing technologies, particularly CRISPR/Cas9, which now enable the development of highly targeted knockout and knock‐in animal models. For example, CRISPR‐engineered leptin receptor–null rats develop obesity and sustained hyperglycemia without the transient remission observed in db/db mice, offering a more stable phenotype for long‐term metabolic studies.^182^ Likewise, Zou et al.^183^ generated a humanized islet amyloid polypeptide (IAPP) miniature pig model using CRISPR/Cas9, successfully replicating key pathological processes such as islet amyloid deposition and β‐cell dysfunction—hallmarks of human T2D that cannot be reproduced in rodent systems. This large‐animal platform substantially enhances translational relevance and provides a powerful system for investigating T2D pathogenesis and associated chronic complications. Although some genetically engineered diabetic strains are now available through specialized repositories and breeding centers, many CRISPR/Cas9‐based models remain custom developed for specific experimental objectives. Recent studies continue to expand the application of CRISPR/Cas9‐based diabetes models, including refined β‐cell–specific gene editing, humanized immune‐system models, and large‐animal platforms with enhanced translational relevance.^184^, ^185^, ^186^, ^187^

In addition to CRISPR‐based approaches, humanized and transgenic mouse models have gained significant traction. These include strains expressing HLA alleles—such as modified NOD lines—or those optimized for human islet transplantation. Such models are particularly valuable for dissecting autoimmune mechanisms and for evaluating human‐specific immune responses and tolerance pathways.

Parallel progress has occurred in dietary modeling, where feeding protocols have been refined to more closely reflect westernized eating patterns. For instance, Stott et al.^75^ proposed a standardized HFD containing 45% kcal from fat—balanced between saturated and unsaturated sources—which reliably induces T2D‐like metabolic disturbances. Moreover, the integration of both high‐fat and high‐sugar components better captures real‐world dietary behaviors compared to fat‐only formulations. Increasing evidence also indicates that sex‐based differences significantly modulate metabolic outcomes, emphasizing the importance of incorporating sex‐specific considerations into dietary model design to improve reproducibility and translational relevance. This further reinforces the need for careful model selection and reporting standards. Ongoing efforts to improve diabetes modeling include protocol standardization, refinement of genetic backgrounds, development of humanized and genome‐edited models, incorporation of sex‐specific analyses, and improved reporting standards, all aimed at reducing variability, enhancing reproducibility, and strengthening translational relevance.

## 6 CONCLUSION

This review has outlined the principal methodologies for the experimental induction of diabetes in laboratory animals, emphasizing that each model replicates distinct aspects of the human disease. Chemical methods—particularly those involving STZ and alloxan—remain widely used due to their relative simplicity, low cost, and rapid induction of β‐cell destruction. However, these approaches primarily mimic insulin deficiency rather than the autoimmune or insulin‐resistant mechanisms underlying human diabetes. In contrast, diet‐induced and genetic models, such as high‐fat feeding and ob/ob mice, more closely recapitulate the progressive insulin resistance and obesity‐related features of T2D. Meanwhile, autoimmune processes relevant to T1D are better modeled using immunological or viral triggers, including Coxsackie virus or NOD mice. Notably, no single model fully captures the multifactorial pathophysiology of human diabetes. As such, most animal models are best suited to investigating specific components of the disease rather than its entirety. Thus, the choice of model should be carefully aligned with the study's objectives, weighing considerations of translational relevance, reproducibility, cost, and technical feasibility. Furthermore, the translational success of diabetes research depends heavily on the selection of appropriate experimental models. Aligning model characteristics with the biological mechanisms and therapeutic targets under investigation can improve the predictive value of preclinical findings, enhance the evaluation of emerging interventions, and ultimately facilitate the development of more effective strategies for diabetes prevention and treatment.

## AUTHOR CONTRIBUTIONS

**Milad Faraji:** Conceptualization; writing – original draft; writing – review and editing. **Maedeh Faraji:** Writing – original draft; writing – review and editing. **Nasrin Kazemipour:** Writing – original draft; writing – review and editing.

## FUNDING INFORMATION

The authors did not receive support from any organization for the submitted work.

## CONFLICT OF INTEREST STATEMENT

The authors report there are no competing interests to declare.

## ETHICAL APPROVAL

This article is a review article and does not contain any new studies involving human participants or animals performed by any of the authors. Therefore, ethical approval and an ethics approval number were not required for this work.

## Data Availability

Data sharing not applicable to this article as no datasets were generated or analysed during the current study.

## References

[1] Mezil SA , Abed BA . Complication of diabetes mellitus. Ann Rom Soc Cell Biol. 2021;25(3):1546‐1556.

[2] Magliano DJ , Boyko EJ , IDF Diabetes Atlas 10th Edition Scientific Committee . IDF Diabetes Atlas. International Diabetes Federation; 2021.

[3] Eizirik DL , Szymczak F , Mallone R . Why does the immune system destroy pancreatic β‐cells but not α‐cells in type 1 diabetes? Nat Rev Endocrinol. 2023;19(7):425‐434. doi:10.1038/s41574-023-00826-3 37072614

[4] Galicia‐Garcia U , Benito‐Vicente A , Jebari S , et al. Pathophysiology of type 2 diabetes mellitus. Int J Mol Sci. 2020;21(17):6275. doi:10.3390/ijms21176275 32872570 PMC7503727

[5] Athanasiadou KI , Paschou SA , Markozannes G , et al. Gestational diabetes mellitus subtypes according to oral glucose tolerance test and pregnancy outcomes. Endocrine. 2025;90:1‐9. doi:10.1007/s12020-025-04329-1 40569563 PMC12464073

[6] Mukherjee P , Roy S , Ghosh D , Nandi S . Role of animal models in biomedical research: a review. Lab Anim Res. 2022;38(1):18. doi:10.1186/s42826-022-00128-1 35778730 PMC9247923

[7] Nath D , Barbhuiya PA , Sen S , Pathak MP . A review on in‐vivo and in‐vitro models of obesity and obesity‐associated co‐morbidities. Endocr Metab Immune Disord Drug Targets. 2025;25(6):458‐478. doi:10.2174/0118715303312932240801073903 39136512

[8] Kaur H , Srivastava N . Exploring the landscape: advancements, challenges and future perspectives in rodent models for biomedical research. Int J Pharm Sci. 2024;2(6):21‐34. doi:10.5281/zenodo.11410069

[9] Fajarwati I , Solihin DD , Wresdiyati T , Batubara I . Self‐recovery in diabetic Sprague Dawley rats induced by intraperitoneal alloxan and streptozotocin. Heliyon. 2023;9(5):e15533. doi:10.1016/j.heliyon.2023.e15533 37159693 PMC10163600

[10] Seok J , Warren HS , Cuenca AG , et al. Genomic responses in mouse models poorly mimic human inflammatory diseases. Proc Natl Acad Sci USA. 2013;110(9):3507‐3512. doi:10.1073/pnas.1222878110 23401516 PMC3587220

[11] Perlman RL . Mouse models of human disease: an evolutionary perspective. Evol Med Public Health. 2016;2016(1):170‐176. doi:10.1093/emph/eow014 27121451 PMC4875775

[12] Zhao Z , Cao J , Niu C , et al. Body temperature is a more important modulator of lifespan than metabolic rate in two small mammals. Nat Metab. 2022;4(3):320‐326. doi:10.1038/s42255-022-00545-5 35288719

[13] Benninger RK , Hodson DJ . New understanding of β‐cell heterogeneity and in situ islet function. Diabetes. 2018;67(4):537‐547. doi:10.2337/dbi17-0040 29559510 PMC5860861

[14] Mbaya JK , Nsonizau DMP , Nsolani NM , Mbuy TZ . Blood glucose reference interval in *Mus musculus* mice. OALib J. 2023;10(6):1‐5. doi:10.4236/oalib.1109988

[15] Wang Z , Yang Y , Xiang X , Zhu Y , Men J , He M . Estimation of the normal range of blood glucose in rats. Wei Sheng Yan Jiu. 2010;39(2):133‐142. 20459020

[16] He Q , Su G , Liu K , et al. Sex‐specific reference intervals of hematologic and biochemical analytes in Sprague–Dawley rats using the nonparametric rank percentile method. PLoS One. 2017;12(12):e0189837. doi:10.1371/journal.pone.0189837 29261747 PMC5738108

[17] Aiello SE , Moses MA , Allen DG . The Merck Veterinary Manual. Merck & Company, Incorporated; 2016.

[18] Gupta RC . Biomarkers in Toxicology. Academic Press; 2019.

[19] Mitruka BM , Rawnsley HM . Clinical Biochemical and Hematological Reference Values in Normal Experimental Animals. Masson Publishing; 1977.

[20] Yu J , Wang J , Zhang Y , et al. Glucose‐responsive insulin patch for the regulation of blood glucose in mice and minipigs. Nat Biomed Eng. 2020;4(5):499‐506. doi:10.1038/s41551-019-0508-y 32015407 PMC7231631

[21] Eames SC , Philipson LH , Prince VE , Kinkel MD . Blood sugar measurement in zebrafish reveals dynamics of glucose homeostasis. Zebrafish. 2010;7(2):205‐213. doi:10.1089/zeb.2009.0640 20515318 PMC2882991

[22] Capiotti KM , Junior RA , Kist LW , Bogo MR , Bonan CD , Da Silva RS . Persistent impaired glucose metabolism in a zebrafish hyperglycemia model. Comp Biochem Physiol B Biochem Mol Biol. 2014;171:58‐65. doi:10.1016/j.cbpb.2014.03.005 24704522

[23] Heckler K , Kroll J . Zebrafish as a model for the study of microvascular complications of diabetes and their mechanisms. Int J Mol Sci. 2017;18(9):2002. doi:10.3390/ijms18092002 28925940 PMC5618651

[24] Cao Y , Chen Q , Liu Y , Jin L , Peng R . Research progress on the construction and application of a diabetic zebrafish model. Int J Mol Sci. 2023;24(6):5195. doi:10.3390/ijms24065195 36982274 PMC10048833

[25] Sen K , Satapathy T . Efficient and safe induction of diabetes in experimental animals: a review on alternative models and techniques. J Lab Anim Res. 2024;3(5):27‐39. doi:10.58803/jlar.v3i5.47

[26] Singh R , Gholipourmalekabadi M , Shafikhani SH . Animal models for type 1 and type 2 diabetes: advantages and limitations. Front Endocrinol. 2024;15:1359685. doi:10.3389/fendo.2024.1359685 PMC10912558 38444587

[27] Qamar F , Sultana S , Sharma M . Animal models for induction of diabetes and its complications. J Diabetes Metab Disord. 2023;22(2):1021‐1028. doi:10.1007/s40200-023-01277-3 37975101 PMC10638335

[28] Araujo J , Paradis A , Mendes J , Petrik S , de Rivera C . Induction of type I diabetes mellitus in beagle dogs using alloxan and streptozotocin. Curr Protoc. 2022;2(11):e580. doi:10.1002/cpz1.580 36326552

[29] Algul S , Ozcelik O . Comprehensive review of animal models in diabetes research using chemical agents. Lab Anim. 2025;59(3):356‐363. doi:10.1177/00236772241296199 39817399

[30] Lenzen S . The mechanisms of alloxan‐and streptozotocin‐induced diabetes. Diabetologia. 2008;51(2):216‐226. doi:10.1007/s00125-007-0886-7 18087688

[31] Thurston DE , Pysz I . Chemistry and Pharmacology of Anticancer Drugs. CRC Press; 2021.

[32] Rakieten N . Studies on the diabetogenic action of streptozotocin (NSC‐37917). Cancer Chemother Res. 1963;29:91‐98. 13990585

[33] Tegegne BA , Mekuria AB , Birru EM . Evaluation of anti‐diabetic and anti‐hyperlipidemic activities of hydro‐alcoholic crude extract of the shoot tips of *Crinum abyssinicum* Hochst. ex A. Rich (amaryllidaceae) in mice. J Exp Pharmacol. 2022;14:27‐41. doi:10.2147/JEP.S335650 35136357 PMC8817951

[34] Furman BL . Streptozotocin‐induced diabetic models in mice and rats. Curr Protoc. 2021;1(4):e78. doi:10.1002/cpz1.78 33905609

[35] Ghasemi A , Jeddi S . Streptozotocin as a tool for induction of rat models of diabetes: a practical guide. EXCLI J. 2023;22:274‐294. doi:10.17179/excli2022-5720 36998708 PMC10043433

[36] Wei L , Lu Y , He S , et al. Induction of diabetes with signs of autoimmunity in primates by the injection of multiple‐low‐dose streptozotocin. Biochem Biophys Res Commun. 2011;412(2):373‐378. doi:10.1016/j.bbrc.2011.07.105 21821007

[37] Tekula S , Khurana A , Anchi P , Godugu C . Withaferin—a attenuates multiple low doses of Streptozotocin (MLD‐STZ) induced type 1 diabetes. Biomed Pharmacother. 2018;106:1428‐1440. doi:10.1016/j.biopha.2018.07.090 30119216

[38] Hikmah N , Shita ADP , Maulana H . Diabetic blood glucose level profile with stratified dose streptozotocin (SD‐STZ) and multi low dose streptozotocin (MLD‐STZ) induction methods. J Trop Life Sci. 2015;5(1):30‐34. doi:10.11594/jtls.05.01.06

[39] Yan L‐J . The nicotinamide/streptozotocin rodent model of type 2 diabetes: renal pathophysiology and redox imbalance features. Biomolecules. 2022;12(9):1225. doi:10.3390/biom12091225 36139064 PMC9496087

[40] Adepoju FO , Sokolova KV , Gette IF , et al. Protective effect of betulin on streptozotocin–nicotinamide‐induced diabetes in female rats. Int J Mol Sci. 2024;25(4):2166. doi:10.3390/ijms25042166 38396842 PMC10888537

[41] Faraji M , Noorbakhsh MF , Kazemipour N , et al. Comparison of the effects of metformin, citral, *Cymbopogon citratus* extract and silver nanoparticles of *Cymbopogon citratus* extract on oxidative stress indices and Nrf2 levels in experimental type 2 diabetes in rats. Animal Model Exp Med. 2025;8:2128‐2138. doi:10.1002/ame2.70017 40325955 PMC12884433

[42] Khatinasab S , Kazemipour N , Noorbakhsh MF , Nazifi S , Faraji M , Ahmadi N . Evaluation of citral and green silver nanoparticles from *Cymbopogon citratus* extract on biochemical profile and Nrf2 gene expression in liver tissue of type 2 diabetic rats. Biomed Res Int. 2025;2025(1):9266092. doi:10.1155/bmri/9266092 41104376 PMC12521785

[43] Niu M , Liu Y , Xiang L , et al. Long‐term case study of a Wuzhishan miniature pig with diabetes. Anim Models Exp Med. 2020;3(1):22‐31. doi:10.1002/ame2.12098 PMC7167240 32318656

[44] Larsen MO , Rolin B . Use of the Göttingen minipig as a model of diabetes, with special focus on type 1 diabetes research. ILAR J. 2004;45(3):303‐313. doi:10.1093/ilar.45.3.303 15229377

[45] Strauss A , Moskalenko V , Tiurbe C , et al. Goettingen minipigs (GMP): comparison of two different models for inducing diabetes. Diabetol Metab Syndr. 2012;4(1):7. doi:10.1186/1758-5996-4-7 22390349 PMC3309977

[46] Lee M‐S , Song K‐D , Yang H‐J , Solis CD , Kim S‐H , Lee W‐K . Development of a type II diabetic mellitus animal model using micropig®. Lab Anim Res. 2012;28(3):205‐208. doi:10.5625/lar.2012.28.3.205 23091521 PMC3469849

[47] Gaur LK , Nepom GT , Lernmark A . Low‐dose streptozotocin induces sustained hyperglycemia in *Macaca nemestrina* . Autoimmunity. 2001;33(2):103‐114. doi:10.3109/08916930108995995 11264789

[48] Graham ML , Mutch LA , Rieke EF , et al. Refining the high‐dose streptozotocin‐induced diabetic non‐human primate model: an evaluation of risk factors and outcomes. Exp Biol Med. 2011;236(10):1218‐1230. doi:10.1258/ebm.2011.011064 21917592

[49] Joyner WL , Mayhan WG , Johnson RL , Phares CK . Microvascular alterations develop in Syrian hamsters after the induction of diabetes mellitus by streptozotocin. Diabetes. 1981;30(2):93‐100. doi:10.2337/diab.30.2.93 6162695

[50] Olsen AS , Sarras MP Jr , Intine RV . Limb regeneration is impaired in an adult zebrafish model of diabetes mellitus. Wound Repair Regen. 2010;18(5):532‐542. doi:10.1111/j.1524-475X.2010.00613.x 20840523 PMC2941236

[51] Dunn JS , McLetchie N . Experimental alloxan diabetes in the rat. Lancet. 1943;242(6265):384‐387. doi:10.1016/S0140-6736(00)87397-3

[52] Macdonald Ighodaro O , Mohammed Adeosun A , Adeboye Akinloye O . Alloxan‐induced diabetes, a common model for evaluating the glycemic‐control potential of therapeutic compounds and plants extracts in experimental studies. Medicina. 2017;53(6):365‐374. doi:10.1016/j.medici.2018.02.001 29548636

[53] Kottaisamy CPD , Raj DS , Prasanth Kumar V , Sankaran U . Experimental animal models for diabetes and its related complications—a review. Lab Anim Res. 2021;37(1):23. doi:10.1186/s42826-021-00101-4 34429169 PMC8385906

[54] Aslam M , Jahan S . Effect of *Caralluma tuberculata* on regulation of carbohydrate metabolizing genes in alloxan‐induced rats. J Ethnopharmacol. 2021;271:113897. doi:10.1016/j.jep.2021.113897 33567306

[55] Gore MSD , Kamble HV , Ghedke MS , Dhananjay M , Narsale M , Ithape S . To Carry out Pharmacological Evaluation of Antidepressant Potential of BOSWELLIA Serrata in ALLOXAN Induced Diabetic Rat. World Journal of Pharmaceutical Research; 2025.

[56] Ahrén B , Sundkvist G . Long‐term effects of alloxan in mice. Int J Pancreatol. 1995;17:197‐201. doi:10.1007/BF02788539 7622942

[57] Bacevic M , Rompen E , Radermecker R , Drion P , Lambert F . Practical considerations for reducing mortality rates in alloxan‐induced diabetic rabbits. Heliyon. 2020;6(6):e04103. doi:10.1016/j.heliyon.2020.e04103 32577551 PMC7305394

[58] Misra M , Aiman U . Alloxan: an unpredictable drug for diabetes induction? Indian J Pharm. 2012;44(4):538‐539. doi:10.4103/0253-7613.99348 PMC3469968 23087526

[59] Kim J‐M . Induction of diabetes mellitus using alloxan in Sprague Dawley rats. Cureus. 2024;16(6):e63359. doi:10.7759/cureus.63359 39070316 PMC11283676

[60] Phares CK . Streptozotocin‐induced diabetes in Syrian hamsters: new model of diabetes mellitus. Experientia. 1980;36(6):681‐682. doi:10.1007/bf01970137 6448163

[61] Wang J , Wan R , Mo Y , Zhang Q , Sherwood LC , Chien S . Creating a long‐term diabetic rabbit model. Exp Diabetes Res. 2010;2010(1):289614. doi:10.1155/2010/289614 21234414 PMC3014707

[62] Benchoula K , Khatib A , Quzwain FMC , et al. Optimization of hyperglycemic induction in zebrafish and evaluation of its blood glucose level and metabolite fingerprint treated with *Psychotria malayana* Jack leaf extract. Molecules. 2019;24(8):1506. doi:10.3390/molecules24081506 30999617 PMC6515116

[63] Paramasivam S , SenthilKumar S . Unveiling the reaction mechanism of co‐reactant electrochemiluminescence behavior of alloxan molecule using tris (2, 2′‐bipyridine) ruthenium (II) as a luminophore and its potential of sensing application. Electrochim Acta. 2022;419:140355. doi:10.1016/j.electacta.2022.140355

[64] Bon EI , Lychkovskaya MA . Diabetes mellitus‐approaches to experimental modeling and molecular marker. Bull Smolensk State Med Acad. 2021;20(2):26‐35. doi:10.37903/vsgma.2021.2.4

[65] Kadota I . Studies on experimental diabetes mellitus, as produced by organic reagents: oxine diabetes and dithizone diabetes. J Lab Clin Med. 1950;35(4):568‐591. doi:10.5555/uri:pii:0022214350900552 15412289

[66] Monago CC , Onwuka F , Osaro E . Effect of combined therapy of diabinese and nicotinic acid on liver enzymes in rabbits with dithizone‐induced diabetes. J Exp Pharmacol. 2010;2:145‐153. doi:10.2147/JEP.S11490 27186100 PMC4863294

[67] Zhautikova S , Abdikadirova KR , Abikenova F , Talaspekova YP , Medvedeva I , Chergizova B . Clinical and laboratory assessment of hormonal and metabolic disorders in experimental animals with alloxan, streptozotocin and dithizone diabetes. Bull Karaganda Univ Biol Med Geogr Ser. 2023;111(3):206‐215. doi:10.31489/2023bmg3/206-215

[68] Halim D , Khalifa K , Awadallah R , El‐Hawary Z , El‐Dessouky E . Serum mineral changes in dithizone‐induced diabetes before and after insulin treatment. Z Ernahrungswiss. 1977;16:22‐26. doi:10.1007/BF02021207 855379

[69] Bhavana A et al., Review On Herbal Approaches And In‐vitro And Invivo Screening Methods Of Anti‐Diabetic Activity. Indo Am. J. P. Sci. 2021;8(6).

[70] Karasawa H , Takaishi K , Kumagae Y . Obesity‐induced diabetes in mouse strains treated with gold thioglucose: a novel animal model for studying β‐cell dysfunction. Obesity. 2011;19(3):514‐521. doi:10.1038/oby.2010.171 20706204

[71] Prashar Y , Patel NJ . Methanolic fruit extract of *Myrica nagi* protects the hypothalamus and attenuates inflammation associated with gold thioglucose‐and high‐fat diet‐induced obesity via various adipokines. J Ayurveda Integr Med. 2023;14(2):100582. doi:10.1016/j.jaim.2022.100582 35842377 PMC10307690

[72] Pandey S , Chmelir T , Chottova Dvorakova M . Animal models in diabetic research—history, presence, and future perspectives. Biomedicine. 2023;11(10):2852. doi:10.3390/biomedicines11102852 PMC10603837 37893225

[73] Bahadoran Z , Mirmiran P , Ghasemi A . Monosodium glutamate (MSG)—induced animal model of type 2 diabetes. Pre‐Clinical Models: Techniques and Protocols. Springer; 2018:49‐65. 10.1007/978-1-4939-8994-2_3 30535681

[74] Wali JA , Jarzebska N , Raubenheimer D , Simpson SJ , Rodionov RN , O'Sullivan JF . Cardio‐metabolic effects of high‐fat diets and their underlying mechanisms—a narrative review. Nutrients. 2020;12(5):1505. doi:10.3390/nu12051505 32455838 PMC7284903

[75] Stott NL , Marino JS . High fat rodent models of type 2 diabetes: from rodent to human. Nutrients. 2020;12(12):3650. doi:10.3390/nu12123650 33261000 PMC7761287

[76] Speakman JR . Use of high‐fat diets to study rodent obesity as a model of human obesity. Int J Obes. 2019;43(8):1491‐1492. doi:10.1038/s41366-019-0363-7 30967607

[77] Skovsø S . Modeling type 2 diabetes in rats using high fat diet and streptozotocin. J Diabetes Investig. 2014;5(4):349‐358. doi:10.1111/jdi.12235 PMC4210077 25411593

[78] Saad MI , Kamel MA , Hanafi MY , Helmy MH , Shehata RR . Effect of sitagliptin and glimepiride on glucose homeostasis and cAMP levels in peripheral tissues of HFD/STZ diabetic rats. Am J Biomed Res. 2014;2(3):52‐60. doi:10.12691/ajbr-2-3-3

[79] Wickramasinghe ASD , Attanayake AP , Kalansuriya P . Biochemical characterization of high fat diet fed and low dose streptozotocin induced diabetic Wistar rat model. J Pharmacol Toxicol Methods. 2022;113:107144. doi:10.1016/j.vascn.2021.107144 34896263

[80] Mašek T , Barišić J , Micek V , Starčević K . Cafeteria diet and high‐fructose rodent models of NAFLD differ in the metabolism of important PUFA and palmitoleic acid without additional influence of sex. Nutrients. 2020;12(11):3339. doi:10.3390/nu12113339 33143061 PMC7692718

[81] Morris JL , Bridson TL , Alim MA , et al. Development of a diet‐induced murine model of diabetes featuring cardinal metabolic and pathophysiological abnormalities of type 2 diabetes. Biol Open. 2016;5(8):1149‐1162. doi:10.1242/bio.016790 27402965 PMC5004603

[82] Riccardi G , Rivellese AA , Giacco R . Role of glycemic index and glycemic load in the healthy state, in prediabetes, and in diabetes. Am J Clin Nutr. 2008;87(1):269S‐274S. doi:10.1093/ajcn/87.1.269S 18175767

[83] Xi S , Yin W , Wang Z , et al. A minipig model of high‐fat/high‐sucrose diet‐induced diabetes and atherosclerosis. Int J Exp Pathol. 2004;85(4):223‐231. doi:10.1111/j.0959-9673.2004.00394.x 15312127 PMC2517483

[84] Zhao Y , Niu M , Jia Y , et al. Establishment of type 2 diabetes mellitus models using streptozotocin after 3 months high‐fat diet in Bama minipigs. Anim Biotechnol. 2023;34(7):2295‐2312. doi:10.1080/10495398.2022.2088548 35749713

[85] Bremer AA , Stanhope KL , Graham JL , et al. Fructose‐fed rhesus monkeys: a nonhuman primate model of insulin resistance, metabolic syndrome, and type 2 diabetes. Clin Transl Sci. 2011;4(4):243‐252. doi:10.1111/j.1752-8062.2011.00298.x 21884510 PMC3170136

[86] Kasim‐Karakas SE , Vriend H , Almario R , Chow LC , Goodman MN . Effects of dietary carbohydrates on glucose and lipid metabolism in golden Syrian hamsters. J Lab Clin Med. 1996;128(2):208‐213. doi:10.1016/s0022-2143(96)90013-x 8765217

[87] Briand F , Thiéblemont Q , Muzotte E , Sulpice T . High‐fat and fructose intake induces insulin resistance, dyslipidemia, and liver steatosis and alters in vivo macrophage‐to‐feces reverse cholesterol transport in hamsters. J Nutr. 2012;142(4):704‐709. doi:10.3945/jn.111.153197 22357742

[88] Kim S‐J , Kim D‐J . Alcoholism and diabetes mellitus. Diabetes Metab J. 2012;36(2):108‐115. doi:10.4093/dmj.2012.36.2.108 22540046 PMC3335891

[89] Gond SP , Panday P , Gupta AK , Rajendiran A , Singh A . In‐vivo models as a system for the investigation and screening of antidiabetic efficacy of certain drugs and substances: a review. Diabetes. 2022;13:15.

[90] Danilo C , Leanza S , Guerra F . Total pancreatectomy: indications, advantages and disadvantages—a review. Maedica. 2019;14(4):391‐396. doi:10.26574/maedica.2019 32153671 PMC7035443

[91] Yamauchi H , Chiba J , Kakizaki K , Maeda M , Miyagawa K , Nakamura R . Effect of islet transplantation on diabetes mellitus after subtotal pancreatectomy in rat a quantitative analysis of islet tissue. Tohoku J Exp Med. 1985;147(4):365‐377. doi:10.1620/tjem.147.365 3938575

[92] Bouwens L , Rooman I . Regulation of pancreatic beta‐cell mass. Physiol Rev. 2005;85(4):1255‐1270. doi:10.1152/physrev.00025.2004 16183912

[93] Bonner‐Weir S , Trent DF , Weir GC . Partial pancreatectomy in the rat and subsequent defect in glucose‐induced insulin release. J Clin Invest. 1983;71(6):1544‐1553. doi:10.1172/jci110910 6134752 PMC370360

[94] Peng X , Rao G , Li X , Tong N , Tian Y , Fu X . Preclinical models for type 1 diabetes mellitus‐a practical approach for research. Int J Med Sci. 2023;20(12):1644‐1661. doi:10.7150/ijms.86566 37859703 PMC10583179

[95] Van der Werf N , Kroese FG , Rozing J , Hillebrands JL . Viral infections as potential triggers of type 1 diabetes. Diabetes Metab Res Rev. 2007;23(3):169‐183. doi:10.1002/dmrr.695 17103489

[96] Carré A , Vecchio F , Flodström‐Tullberg M , You S , Mallone R . Coxsackievirus and type 1 diabetes: diabetogenic mechanisms and implications for prevention. Endocr Rev. 2023;44(4):737‐751. doi:10.1210/endrev/bnad007 36884282

[97] Tanveer A . Using Immunosignatures to Identify LCMV Infection and an Investigation into Autoimmunity in a Mouse Model of Noonan Syndrome. Arizona State University; 2021.

[98] Burke RM , Tate JE , Jiang B , Parashar UD . Rotavirus and type 1 diabetes—is there a connection? A synthesis of the evidence. J Infect Dis. 2020;222(7):1076‐1083. doi:10.1093/infdis/jiaa168 32249284 PMC8112884

[99] Wu L , Tsang VH , Sasson SC , et al. Unravelling checkpoint inhibitor associated autoimmune diabetes: from bench to bedside. Front Endocrinol. 2021;12:764138. doi:10.3389/fendo.2021.764138 PMC8603930 34803927

[100] Coppieters KT , Boettler T , von Herrath M . Virus infections in type 1 diabetes. Cold Spring Harb Perspect Med. 2012;2(1):a007682. doi:10.1101/cshperspect.a007682 22315719 PMC3253029

[101] Aldrich VR , Hernandez‐Rovira BB , Chandwani A , Abdulreda MH . NOD mice‐good model for T1D but not without limitations. Cell Transplant. 2020;29:963689720939127. doi:10.1177/0963689720939127 32762460 PMC7563935

[102] Standley A , Xie J , Lau AW , Grote L , Gifford AJ . Working with miraculous mice: *Mus musculus* as a model organism. Curr Protoc. 2024;4(10):e70021. doi:10.1002/cpz1.70021 39435766

[103] Bortell R , Yang C . The BB rat as a model of human type 1 diabetes. Animal Models in Diabetes Research. Springer; 2012:31‐44. 10.1007/978-1-62703-068-7_3 22893399

[104] Hornum L , Rømer J , Markholst H . The diabetes‐prone BB rat carries a frameshift mutation in Ian4, a positional candidate of Iddm1. Diabetes. 2002;51(6):1972‐1979. doi:10.2337/diabetes.51.6.1972 12031988

[105] Lenzen S , Arndt T , Elsner M , Wedekind D , Jörns A . Rat models of human type 1 diabetes. Methods Mol Biol. 2020;2128:69‐85. doi:10.1007/978-1-0716-0385-7_5 32180186

[106] Lenzen S , Tiedge M , Elsner M , et al. The LEW.1AR1/Ztm‐iddm rat: a new model of spontaneous insulin‐dependent diabetes mellitus. Diabetologia. 2001;44(9):1189‐1196. doi:10.1007/s001250100625 11596676

[107] Yagihashi S . Contribution of animal models to diabetes research: its history, significance, and translation to humans. J Diabetes Investig. 2023;14(9):1015‐1037. doi:10.1111/jdi.14034 PMC10445217 37401013

[108] Gvazava IG , Karimova MV , Vasiliev AV , Vorotelyak EA . Type 2 diabetes mellitus: pathogenic features and experimental models in rodents. Acta Nat. 2022;14(3):57‐68. doi:10.32607/actanaturae.11751 PMC9611859 36348712

[109] Suriano F , Vieira‐Silva S , Falony G , et al. Novel insights into the genetically obese (ob/ob) and diabetic (db/db) mice: two sides of the same coin. Microbiome. 2021;9(1):147. doi:10.1186/s40168-021-01097-8 34183063 PMC8240277

[110] Fang X , Du Z , Duan C , et al. Beinaglutide shows significantly beneficial effects in diabetes/obesity‐induced nonalcoholic steatohepatitis in ob/ob mouse model. Life Sci. 2021;270:118966. doi:10.1016/j.lfs.2020.118966 33482185

[111] Berger C , Heyne HO , Heiland T , et al. A novel compound heterozygous leptin receptor mutation causes more severe obesity than in Lepr^db/db^ mice. J Lipid Res. 2021;62:100105. doi:10.1016/j.jlr.2021.100105 34390703 PMC8450258

[112] Fajardo RJ , Karim L , Calley VI , Bouxsein ML . A review of rodent models of type 2 diabetic skeletal fragility. J Bone Miner Res. 2014;29(5):1025‐1040. doi:10.1002/jbmr.2210 24585709 PMC5315418

[113] Zhao S , Yu Q . Principles of creating and using animal models for studying human diseases. Fundamentals of Laboratory Animal Science. CRC Press; 2017:189‐222.

[114] Davis RC , Castellani LW , Hosseini M , et al. Early hepatic insulin resistance precedes the onset of diabetes in obese C57BLKS‐db/db mice. Diabetes. 2010;59(7):1616‐1625. doi:10.2337/db09-0878 20393148 PMC2889760

[115] Lutz TA . An overview of rodent models of obesity and type 2 diabetes. In: King A , eds. Animal Models of Diabetes: Methods in Molecular Biology; 2020:11‐24. 10.1007/978-1-0716-0385-7_2 32180183

[116] Al‐Awar A , Kupai K , Veszelka M , et al. Experimental diabetes mellitus in different animal models. J Diabetes Res. 2016;2016:9051426. doi:10.1155/2016/9051426 27595114 PMC4993915

[117] Han L , Bittner S , Dong D , et al. Molecular changes in hepatic metabolism in ZDSD rats—a new polygenic rodent model of obesity, metabolic syndrome, and diabetes. Biochim Biophys Acta Mol Basis Dis. 2020;1866(5):165688. doi:10.1016/j.bbadis.2020.165688 31987840 PMC8229451

[118] Wang AN , Carlos J , Fraser GM , JJ MG . Zucker diabetic‐Sprague Dawley (ZDSD) rat: type 2 diabetes translational research model. Exp Physiol. 2022;107(4):265‐282. doi:10.1113/EP089947 35178802 PMC9314054

[119] GoTo Y , Kakizaki M , Masaki N . Spontaneous diabetes produced by selective breeding of normal Wistar rats. Proc Jpn Acad. 1975;51(1):80‐85. doi:10.2183/pjab1945.51.80

[120] Movassat J , Saulnier C , Serradas P , Portha B . Impaired development of pancreatic beta‐cell mass is a primary event during the progression to diabetes in the GK rat. Diabetologia. 1997;40(8):916‐925. doi:10.1007/s001250050768 9267986

[121] Seal SV , Henry M , Pajot C , et al. A holistic view of the goto‐kakizaki rat immune system: decreased circulating immune markers in non‐obese type 2 diabetes. Front Immunol. 2022;13:896179. doi:10.3389/fimmu.2022.896179 35677049 PMC9168276

[122] Nugent DA , Smith DM , Jones HB . A review of islet of Langerhans degeneration in rodent models of type 2 diabetes. Toxicol Pathol. 2008;36(4):529‐551. doi:10.1177/0192623308318209 18467681

[123] Wang X . Analysis of Transition from Pre‐Diabetes to Diabetes in Two Animal Models and Evaluation of Anti‐Diabetic Effects of Salsalate. State University of New York at Buffalo; 2014.

[124] ZhePeng W , WenXin z , JunXi H , QiaoYan H , JiaYue Z . Association of levels of cholecystokinin a receptor expression and sequence variants with feed conversion efficiency of Lueyang black‐boned chicken. Sci Agric Sin. 2022;55(22):4539‐4549. doi:10.3864/j.issn.0578-1752.2022.22.017

[125] Chowdhury NN , Surowiec RK , Kohler RK , Reul ON , Segvich DM , Wallace JM . Metabolic and skeletal characterization of the KK/ay mouse model—a polygenic mutation model of obese type 2 diabetes. Calcif Tissue Int. 2024;114(6):638‐649. doi:10.1007/s00223-024-01216-1 38642089 PMC11184323

[126] Oki C , Uno K , Sasase T , et al. High‐fat/high‐sucrose diet‐induced renal changes in obese diabetic mice: a comparison with db/db and KK‐ay mice. J Vet Med Sci. 2025;87(2):138‐146. doi:10.1292/jvms.24-0313 39662940 PMC11830451

[127] Pasieka AM , Rafacho A . Impact of glucocorticoid excess on glucose tolerance: clinical and preclinical evidence. Metabolites. 2016;6(3):24. doi:10.3390/metabo6030024 27527232 PMC5041123

[128] van Raalte DH . Diabetogenic effects of glucocorticoid drugs: the knowns and the unknowns. Eur J Clin Investig. 2012;39:81‐93. doi:10.1111/j.1365-2362.2008.02067.x 19200161

[129] Niu L , Chen Q , Hua C , et al. Effects of chronic dexamethasone administration on hyperglycemia and insulin release in goats. J Anim Sci Biotechnol. 2018;9(1):26. doi:10.1186/s40104-018-0242-4 29568520 PMC5855938

[130] Alsaedi AQ , Nader MA , El‐Kashef DH , Abdelmageed ME . Mangiferin mitigates dexamethasone‐induced insulin resistance in rats: insight into vascular dysfunction and hepatic steatosis. Front Pharmacol. 2025;16:1572758. doi:10.3389/fphar.2025.1572758 40406487 PMC12095298

[131] Beaupere C , Liboz A , Fève B , Blondeau B , Guillemain G . Molecular mechanisms of glucocorticoid‐induced insulin resistance. Int J Mol Sci. 2021;22(2):623. doi:10.3390/ijms22020623 33435513 PMC7827500

[132] Shpilberg Y , Beaudry JL , D'Souza A , Campbell JE , Peckett A , Riddell MC . A rodent model of rapid‐onset diabetes induced by glucocorticoids and high‐fat feeding. Dis Model Mech. 2012;5(5):671‐680. doi:10.1242/dmm.008912 22184636 PMC3424464

[133] Gathercole L , Morgan S , Bujalska I , Stewart P , Tomlinson J . Short‐and long‐term glucocorticoid treatment enhances insulin signalling in human subcutaneous adipose tissue. Nutr Diabetes. 2011;1(1):e3. doi:10.1038/nutd.2010.3 23154295 PMC3302128

[134] Fransson L , Franzén S , Rosengren V , Wolbert P , Sjöholm Å , Ortsäter H . B‐cell adaptation in a mouse model of glucocorticoid‐induced metabolic syndrome. J Endocrinol. 2013;219(3):231‐241. doi:10.1530/JOE-13-0189 24048967

[135] Heidt DG , Burant C , Simeone DM . Total pancreatectomy: indications, operative technique, and postoperative sequelae. J Gastrointest Surg. 2007;11(2):209‐216. doi:10.1007/s11605-006-0025-7 17390175

[136] Scholten L , Stoop T , Del Chiaro M , et al. Systematic review of functional outcome and quality of life after total pancreatectomy. Br J Surg. 2019;106(13):1735‐1746. doi:10.1002/bjs.11296 31502658 PMC6899549

[137] Khan A . Total Pancreatectomy for Chronic Pancreatitis: Surgical Technique and Recent Advances. In: Oviedo RJ , eds. Upper Gastrointestinal Surgery ‐ Contemporary Techniques and Training. IntechOpen; 2025.

[138] Thisted L , Østergaard MV , Pedersen AA , et al. Rat pancreatectomy combined with isoprenaline or uninephrectomy as models of diabetic cardiomyopathy or nephropathy. Sci Rep. 2020;10(1):16130. doi:10.1038/s41598-020-73046-8 32999377 PMC7527487

[139] Jin X , Zeng L , He S , et al. Comparison of single high‐dose streptozotocin with partial pancreatectomy combined with low‐dose streptozotocin for diabetes induction in rhesus monkeys. Exp Biol Med. 2010;235(7):877‐885. doi:10.1258/ebm.2010.009361 20558842

[140] Li L , Seno M , Yamada H , Kojima I . Promotion of beta‐cell regeneration by betacellulin in 90%‐pancreatectomized rats. Endocrinology. 2001;142(12):5379‐5385. doi:10.1210/endo.142.12.8520 11713238

[141] Fathi I , Imura T , Imura K , et al. A simple and effective non‐human primate diabetic model combining sub‐total pancreatectomy and low‐dose streptozotocin injection. OBM Transplant. 2019;3(3):1‐23. doi:10.21926/obm.transplant.1903071

[142] Esteves‐Monteiro M , Ferreira‐Duarte M , Vitorino‐Oliveira C , et al. Oxidative stress and Histomorphometric remodeling: two key intestinal features of type 2 diabetes in Goto–Kakizaki rats. Int J Mol Sci. 2024;25(22):12115. doi:10.3390/ijms252212115 39596183 PMC11594829

[143] Babaya N , Nakayama M , Moriyama H , et al. A new model of insulin‐deficient diabetes: male NOD mice with a single copy of Ins1 and no Ins2. Diabetologia. 2006;49(6):1222‐1228. doi:10.1007/s00125-006-0241-4 16612590

[144] Guo C , Zhang S , Li J‐Y , et al. Chronic hyperglycemia induced via the heterozygous knockout of Pdx1 worsens neuropathological lesion in an Alzheimer mouse model. Sci Rep. 2016;6(1):29396. doi:10.1038/srep29396 27406855 PMC4942607

[145] Zhang Y , Fang X , Wei J , et al. PDX‐1: a promising therapeutic target to reverse diabetes. Biomolecules. 2022;12(12):1785. doi:10.3390/biom12121785 36551213 PMC9775243

[146] Aubin A‐M , Lombard‐Vadnais F , Collin R , Aliesky HA , McLachlan SM , Lesage S . The NOD mouse beyond autoimmune diabetes. Front Immunol. 2022;13:874769. doi:10.3389/fimmu.2022.874769 35572553 PMC9102607

[147] Engerman RL , Kramer JW . Dogs with induced or spontaneous diabetes as models for the study of human diabetes mellitus. Diabetes. 1982;31(suppl. 1):26‐29. doi:10.2337/diab.31.1.S26 6761192

[148] Adin CA , Gilor C . The diabetic dog as a translational model for human islet transplantation. Yale J Biol Med. 2017;90(3):509‐515. 28955189 PMC5612193

[149] Hirose M , Inoue K , Matoba S , et al. Disruption of insulin receptor substrate 2 (IRS2) causes non‐obese type 2 diabetes with β‐cell dysfunction in the golden (Syrian) hamster. Sci Rep. 2024;14(1):17450. doi:10.1038/s41598-024-67513-9 39134590 PMC11319448

[150] Kimmel RA , Dobler S , Schmitner N , Walsen T , Freudenblum J , Meyer D . Diabetic pdx1‐mutant zebrafish show conserved responses to nutrient overload and anti‐glycemic treatment. Sci Rep. 2015;5(1):14241. doi:10.1038/srep14241 26384018 PMC4585597

[151] Wiggenhauser LM , Metzger L , Bennewitz K , et al. Pdx1 knockout leads to a diabetic nephropathy—like phenotype in zebrafish and identifies phosphatidylethanolamine as metabolite promoting early diabetic kidney damage. Diabetes. 2022;71(5):1073‐1080. doi:10.2337/db21-0645 35100334

[152] Zhou Q , Li M , Zhang J , et al. PPARγ activation reduces pancreatic beta cell death in type 1 diabetes by decreasing heparanase‐dependent insulitis. Int Immunopharmacol. 2025;162:115183. doi:10.1016/j.intimp.2025.115183 40628043

[153] Lin C‐H , Kuo Y‐H , Shih C‐C . Antidiabetic and immunoregulatory activities of extract of *Phyllanthus emblica* L. in NOD with spontaneous and cyclophosphamide‐accelerated diabetic mice. Int J Mol Sci. 2023;24(12):9922. doi:10.3390/ijms24129922 37373070 PMC10298654

[154] Yang SJ , Singh AK , Drow T , et al. Pancreatic islet‐specific engineered Tregs exhibit robust antigen‐specific and bystander immune suppression in type 1 diabetes models. Sci Transl Med. 2022;14(665):eabn1716. doi:10.1126/scitranslmed.abn1716 36197963

[155] Oulghazi S , Wegner SK , Spohn G , et al. Adaptive immunity and pathogenesis of diabetes: insights provided by the α4–integrin deficient NOD mouse. Cells. 2020;9(12):2597. doi:10.3390/cells9122597 33291571 PMC7761835

[156] Sun J , Shi J , Li J , et al. The effect of immunosuppressive adjuvant kynurenine on type 1 diabetes vaccine. Front Immunol. 2021;12:681328. doi:10.3389/fimmu.2021.681328 34305913 PMC8293994

[157] Elagin RB , Jaume JC . Glucose intolerance and diabetes following antigen‐specific insulitis in diabetes‐susceptible “humanized” transgenic mice. Biochem Biophys Res Commun. 2010;395(1):99‐103. doi:10.1016/j.bbrc.2010.03.146 20350527 PMC2906138

[158] Karges W , Pechhold K , Al Dahouk S , et al. Induction of autoimmune diabetes through insulin (but not GAD65) DNA vaccination in nonobese diabetic and in RIP‐B7. 1 mice. Diabetes. 2002;51(11):3237‐3244. doi:10.2337/diabetes.51.11.3237 12401715

[159] Perdigoto AL , Deng S , Du KC , et al. Immune cells and their inflammatory mediators modify β cells and cause checkpoint inhibitor–induced diabetes. JCI Insight. 2022;7(17):e156330. doi:10.1172/jci.insight.156330 35925682 PMC9536276

[160] Collier JL , Pauken KE , Lee CA , et al. Single‐cell profiling reveals unique features of diabetogenic T cells in anti‐PD‐1‐induced type 1 diabetes mice. J Exp Med. 2023;220(10):e20221920. doi:10.1084/jem.20221920 37432393 PMC10336233

[161] Mine K , Yoshikai Y , Takahashi H , Mori H , Anzai K , Nagafuchi S . Genetic susceptibility of the host in virus‐induced diabetes. Microorganisms. 2020;8(8):1133. doi:10.3390/microorganisms8081133 32727064 PMC7464158

[162] Sundaresan B , Shirafkan F , Ripperger K , Rattay K . The role of viral infections in the onset of autoimmune diseases. Viruses. 2023;15(3):782. doi:10.3390/v15030782 36992490 PMC10051805

[163] Christoffersson G , Flodström‐Tullberg M . Mouse models of virus‐induced type 1 diabetes. Methods Mol Biol. 2020;2128:93‐105. doi:10.1007/978-1-0716-0385-7_7 32180188

[164] Yoon JW , Jun HS . Viruses cause type 1 diabetes in animals. Ann N Y Acad Sci. 2006;1079:138‐146. doi:10.1196/annals.1375.021 17130545

[165] Stone VM , Butrym M , Hankaniemi MM , et al. Coxsackievirus B vaccines prevent infection‐accelerated diabetes in NOD mice and have no disease‐inducing effect. Diabetes. 2021;70(12):2871‐2878. doi:10.2337/db21-0193 34497136 PMC8660981

[166] Rogowicz‐Frontczak A , Majchrzak A , Zozulińska‐Ziółkiewicz D . Insulin resistance in endocrine disorders—treatment options. Endokrynol Pol. 2017;68(3):334‐351. doi:10.5603/EP.2017.0026 28660991

[167] Olsson B , Bohlooly YM , Fitzgerald SM , et al. Bovine growth hormone transgenic mice are resistant to diet‐induced obesity but develop hyperphagia, dyslipidemia, and diabetes on a high‐fat diet. Endocrinology. 2005;146(2):920‐930. doi:10.1210/en.2004-1232 15539551

[168] Ali S , Drucker DJ . Benefits and limitations of reducing glucagon action for the treatment of type 2 diabetes. Am J Physiol Endocrinol Metab. 2009;296(3):E415‐E421. doi:10.1152/ajpendo.90887.2008 19116373

[169] Rodgers RL . Glucagon, cyclic AMP, and hepatic glucose mobilization: a half‐century of uncertainty. Physiol Rep. 2022;10(9):e15263. doi:10.14814/phy2.15263 35569125 PMC9107925

[170] Yokota‐Nakagi N , Omoto S , Tazumi S , Kawakami M , Takamata A , Morimoto K . Estradiol replacement improves high‐fat diet‐induced insulin resistance in ovariectomized rats. Physiol Rep. 2022;10(5):e15193. doi:10.14814/phy2.15193 35238495 PMC8892597

[171] Riant E , Waget A , Cogo H , Arnal J‐F , Burcelin R , Gourdy P . Estrogens protect against high‐fat diet‐induced insulin resistance and glucose intolerance in mice. Endocrinology. 2009;150(5):2109‐2117. doi:10.1210/en.2008-0971 19164473

[172] Atkinson MA , Eisenbarth GS , Michels AW . Type 1 diabetes. Lancet. 2014;383(9911):69‐82. doi:10.1016/S0140-6736(13)60591-7 23890997 PMC4380133

[173] Eizirik DL , Colli ML , Ortis F . The role of inflammation in insulitis and β‐cell loss in type 1 diabetes. Nat Rev Endocrinol. 2009;5(4):219‐226. doi:10.1038/nrendo.2009.21 19352320

[174] Coppieters KT , Dotta F , Amirian N , et al. Demonstration of islet‐autoreactive CD8 T cells in insulitic lesions from recent onset and long‐term type 1 diabetes patients. J Exp Med. 2012;209(1):51‐60. doi:10.1084/jem.20111187 22213807 PMC3260877

[175] Kandinov I , Knyazeva A , Lander E , Gryadunov D , Savvateeva E . Molecular portrait of autoantigens in type 1 diabetes. Biomolecules. 2025;15(12):1723. doi:10.3390/biom15121723 41463376 PMC12730728

[176] Campbell‐Thompson M , Fu A , Kaddis JS , et al. Insulitis and β‐cell mass in the natural history of type 1 diabetes. Diabetes. 2016;65(3):719‐731. doi:10.2337/db15-0779 26581594 PMC4764143

[177] Pearson JA , Wong FS , Wen L . The importance of the non obese diabetic (NOD) mouse model in autoimmune diabetes. J Autoimmun. 2016;66:76‐88. doi:10.1016/j.jaut.2015.08.019 26403950 PMC4765310

[178] Atamni HJA‐T , Mott R , Soller M , Iraqi FA . High‐fat‐diet induced development of increased fasting glucose levels and impaired response to intraperitoneal glucose challenge in the collaborative cross mouse genetic reference population. BMC Genet. 2016;17(1):10. doi:10.1186/s12863-015-0321-x 26728312 PMC4700737

[179] Srinivasan K , Ramarao P . Animal models in type 2 diabetes research: an overview. Indian J Med Res. 2007;125(3):451‐472. doi:10.25259/IJMR_20071253_451 17496368

[180] Kumar S , Singh R , Vasudeva N , Sharma S . Acute and chronic animal models for the evaluation of anti‐diabetic agents. Cardiovasc Diabetol. 2012;11(1):9. doi:10.1186/1475-2840-11-9 22257465 PMC3286385

[181] Hogh K‐LN . Characterization of PPAR‐Alpha Overexpression in Beta‐Cell Lipotoxicity and its Effects on Obesity‐Induced Type 2 Diabetes. University of Northern British Columbia; 2012.

[182] Bao D , Ma Y , Zhang X , et al. Preliminary characterization of a leptin receptor knockout rat created by CRISPR/Cas9 system. Sci Rep. 2015;5(1):15942. doi:10.1038/srep15942 26537785 PMC4633582

[183] Zou X , Ouyang H , Yu T , et al. Preparation of a new type 2 diabetic miniature pig model via the CRISPR/Cas9 system. Cell Death Dis. 2019;10(11):823. doi:10.1038/s41419-019-2056-5 31659151 PMC6817862

[184] Russo F , Ruggiero E , Curto R , et al. Editing T cell repertoire by thymic epithelial cell‐directed gene transfer abrogates risk of type 1 diabetes development. Mol Ther Methods Clin Dev. 2022;25:508‐519. doi:10.1016/j.omtm.2022.04.017 35615710 PMC9121074

[185] Peters LD , Yeh W‐I , Arnoletti JM , et al. Modeling cell‐mediated immunity in human type 1 diabetes by engineering autoreactive CD8+ T cells. Front Immunol. 2023;14:1142648. doi:10.3389/fimmu.2023.1142648 37325626 PMC10262917

[186] Shah P . Genomic Editing and Diabetes. Genome Editing in Cardiovascular and Metabolic Diseases. Springer; 2022:207‐214.

[187] Lotfi M , Butler AE , Sukhorukov VN , Sahebkar A . Application of CRISPR‐Cas9 technology in diabetes research. Diabet Med. 2024;41(1):e15240. doi:10.1111/dme.15240 37833064

